# FairEdge360: Distributed Multi-Agent Reinforcement Learning for QoE-Fair 360° Video Streaming with Uncertainty-Aware Edge Coordination

**DOI:** 10.3390/jimaging12060234

**Published:** 2026-05-28

**Authors:** Reka Sandaruwan Gallena Watthage, Anil Fernando

**Affiliations:** Department of Computer & Information Sciences, University of Strathclyde, Glasgow G1 1XH, UK; anil.fernando@strath.ac.uk

**Keywords:** 360-degree video streaming, multi-agent reinforcement learning, quality of experience, viewport prediction, edge computing, fairness, graph neural networks, uncertainty estimation

## Abstract

Shared immersive environment sports venues, virtual classrooms, and collaborative workspaces require multiple users to stream 360° videos simultaneously over the same edge network, yet every existing adaptive bitrate system optimises each viewer in isolation. This self-interested behaviour triggers a bandwidth auction that chronically starves the most uncertain viewers: Jain’s Fairness Index for ten independently optimised agents routinely falls below 0.85. We present FairEdge360, a hierarchical multi-agent reinforcement learning framework that reformulates multi-user 360° streaming as a Decentralised Partially Observable Markov Decision Process (Dec-POMDP) and proves, formally, that fairness and quality are complementary rather than competing objectives. Three tightly coupled innovations make this possible. First, a Lightweight Uncertainty Estimator (LUE) a compact 8385-parameter four-layer MLP evaluates per-device viewport prediction confidence $c_{t}^{i}=\sigma \left(w_{4}^{⊤}h_{3}\right)$ in under approximately 2.1 ms on commodity smartphones (95th percentile, iPhone 12 A14 Bionic), enabling selective edge offloading that reduces device energy consumption by 38.9%. Second, a variational Graph Neural Network compresses each agent’s 256-dimensional GRU state into a 32-byte INT8 latent, transmitted over a dynamic RTT-gated neighbourhood graph at 96 bytes per agent per 500 ms 75% less overhead than competing approaches. Third, the edge coordinator maximises the Nash social welfare objective $\mathrm{NSW}={\left(\prod_{i=1}^{N}Q_{i}\right)}^{1/N}$, whose gradient $\partial \;\mathrm{NSW}/\partial Q_{i}\propto 1/Q_{i}$ automatically prioritises the most disadvantaged viewer; a formal proof guarantees that every Pareto-optimal policy satisfies $Q_{i}/\sum_{j}Q_{j}\geq 1/N$. Counterfactual advantage estimation correctly attributes each agent’s marginal contribution to the global reward, eliminating the credit-assignment ambiguity inherent in standard multi-agent baselines. Evaluated on 284 users, 52 omnidirectional videos, and 10,000 real network traces spanning 4G LTE, 5G mmWave, HSDPA, and campus WiFi, FairEdge360 raises Jain’s Fairness Index from 0.934 to 0.976 (+4.5%), improves worst-case user quality-of-experience from MOS 2.54 to MOS 3.21 (+26.4%), and halves rebuffering rate from 2.1% to 1.1%, all within a 20 ms motion-to-photon budget on a commodity smartphone.

## 1. Introduction

Shared immersive environment sports venues, virtual classrooms, and collaborative workspaces require multiple users to stream 360° video simultaneously over the same edge network. Yet every existing adaptive bitrate (ABR) system optimises each viewer in isolation, triggering a bandwidth auction that chronically disadvantages the most uncertain users: Jain’s Fairness Index for ten independently optimised streaming agents in a bandwidth-constrained shared network routinely falls below 0.85 [1]. The global virtual reality market, projected to exceed USD 435 billion by 2032 [2], makes equitable multi-user streaming an increasingly urgent engineering challenge.

Streaming omnidirectional content faithfully over best-effort networks is harder than conventional video delivery: a single 4K equirectangular frame encodes up to 36 megapixels, yet only roughly 20% falls within any given viewer’s 110°-wide field of view at any instant [3]. The community has responded with viewport-adaptive streaming partitioning the sphere into independently coded tiles and allocating bitrate proportional to predicted viewport probability [4,5] and a progression of viewport predictors from sinusoidal heuristics [6,7] through recurrent networks [8,9] to transformers and multi-modal attention [10,11,12,13,14]. Adaptive bitrate algorithms have incorporated stochastic optimisation under uncertain viewports and bandwidth [15,16,17], and reinforcement learning has proved effective for sequential streaming decisions [18,19,20]. Comprehensive surveys and benchmark datasets have further accelerated progress [21,22,23,24,25,26]. Critically, every one of these contributions optimises the experience of a single user in isolation, with no mechanism for coordinated allocation across a shared link.

Recent multi-user frameworks have begun to close this gap. Liu et al. [1] proposed STMRQ, combining a spatio-temporal graph convolutional network with multi-agent deep reinforcement learning for QoE fairness. Wang et al. [27] developed CoLive, an edge-assisted collaborative learning framework for concurrent viewers. Nguyen Viet et al. [28] introduced COSMN, a clustering-based optimisation for live 360° streaming over mobile networks. However, none of these systems explicitly quantifies viewport prediction uncertainty at the per-agent level, none combines uncertainty-aware on-device inference with a game-theoretic fairness objective, and none provides tight communication efficiency guarantees an essential requirement when GNN-based coordination risks consuming more overhead bandwidth than it saves in quality gains.

We present FairEdge360, a hierarchical multi-agent reinforcement learning framework that addresses all three gaps simultaneously. FairEdge360 treats multi-user 360° streaming as a Decentralised Partially Observable Markov Decision Process (Dec-POMDP), with each device hosting a lightweight agent that makes local decisions while exchanging compressed latent representations with neighbours via a variational Graph Neural Network (GNN). A Lightweight Uncertainty Estimator (LUE) a compact 8385-parameter neural network runs continuously on each device, estimating viewport predictor confidence and triggering selective edge offloading only when uncertainty is genuinely high. The edge coordinator enforces a global bandwidth constraint and allocates computation preferentially to uncertain agents under the Nash social welfare objective the geometric mean of individual QoE scores. Evaluated on 284 users, 52 videos, and 10,000 network traces spanning 4G, 5G, HSDPA, and WiFi, FairEdge360 achieves a Jain’s Fairness Index of 0.976 (+4.5% over STMRQ), Nash social welfare of 3.95 (+9.1%), and worst-case QoE of 3.21 (+26.4%), while reducing communication overhead by 75%.

The principal contributions of this paper are:

1. FairEdge360 framework: A hierarchical Dec-POMDP formulation for multi-user 360° streaming that jointly optimises fairness, viewport quality, and communication efficiency across up to 15 concurrent users (Section 3).
2. Lightweight Uncertainty Estimator (LUE): An 8385-parameter on-device network that predicts viewport prediction confidence in 2.1–3.4 ms, enabling selective edge offloading and uncertainty-proportional resource allocation with a 38.9% reduction in device energy consumption.
3. Nash social welfare with counterfactual advantage: A reward formulation whose gradient $\partial R^{\mathrm{global}}/\partial Q_{i}\propto 1/Q_{i}$ automatically prioritises the most disadvantaged viewer, combined with a counterfactual credit assignment mechanism that correctly attributes each agent’s marginal contribution to global QoE.
4. Communication-efficient GNN coordination: A variational GNN that compresses agent state into 32-byte INT8-quantised latents, reducing inter-agent overhead to approximately 240 bytes per agent per 500 ms decision epoch 75% less than full-state sharing.

The remainder of this paper is organised as follows. Section 2 reviews related work across viewport prediction, multi-user adaptive streaming, multi-agent reinforcement learning, fairness mechanisms, and edge-assisted streaming. Section 3 presents the FairEdge360 architecture, Dec-POMDP formulation, and training algorithm in full detail. Section 4 describes the experimental setup, datasets, baselines, and evaluation protocols. Section 5 presents and analyses the results. Section 6 discusses implications, limitations, and directions for future work. Section 7 concludes the paper.

## 2. Related Work

Research in 360° video streaming has progressively improved viewport prediction accuracy, bitrate adaptation, and quality metrics over the past decade. However, the multi-user fairness problem has received comparatively little attention until recently. This section reviews the foundations on which FairEdge360 builds and identifies the gaps that motivated its design.

### 2.1. Viewport Prediction

Early viewport predictors used sinusoidal fits to head rotation trajectories [6,7], which are cheap and competitive at short horizons but break down under non-linear head movements and provide no prediction confidence information. Recurrent architectures improved substantially on these heuristics: Xu et al. [8] showed that LSTMs trained on head-motion histories outperform extrapolation beyond 0.5 s, and Martinez et al. [29] provided foundational insights into GRU-based sequence-to-sequence motion modelling subsequently adapted to head-motion prediction [30,31]. Hu et al. [9] proposed a reinforcement-learning formulation, framing viewport selection as a deep agent tracking salient objects in 360° sports video.

Content-based and transformer architectures then became dominant. PARIMA [32] combines scene-level saliency estimation with head-motion refinement; STAR-VP [33] applies space-aligned and time-varying feature fusion; and VPT360 [10], MFTR [11], and CMMST [12] demonstrate that scanpath-attention and cross-modal transformer architectures consistently surpass CNN-RNN hybrids in both accuracy and efficiency. The EMD-ML approach of Zhou et al. [34] exploits multi-scale empirical mode decomposition to achieve nearly a 50% reduction in orthodromic distance, while MDA-based attention [14] and bidirectional transformer alignment [13] further refine long-horizon predictions. Benchmark datasets from Wu et al. [23] (48 users, 18 videos), Corbillon et al. [24] (59 users, 5 videos), David et al. [25] (57 users, eye-tracking), Rai et al. [26], and Xu et al. [35] (120-Hz head and eye tracking) have underpinned this progress. TRACK [36] provides the most thorough architectural ablation in the literature; its findings directly inform FairEdge360’s Bayesian predictor backbone. A comprehensive review by Wahba et al. [22] identifies cross-content generalisation and per-user personalisation as the two most persistent open problems.

For live streaming, Gaussian mixture model approaches [37] and mobile-optimised predictors [38] have shown practical feasibility. For 6DoF volumetric content, Li et al. [39] and Setayesh and Wong [40] address the coupling between viewport prediction and physical-layer resource management in multicast and THz scenarios respectively. Transformer architectures have extended beyond viewport prediction into video compression and enhancement: recent work [41] demonstrates that cross-attention over spatial-temporal feature hierarchies substantially improves perceptual quality under bandwidth constraints, motivating the attention-based design of FairEdge360’s MDN covariance head.

Essentially every method above models a single user, with no concept of how viewport predictions interact across concurrent users sharing bandwidth, and no predictor integrates uncertainty quantification directly into a multi-user resource allocation loop. FairEdge360 addresses both gaps.

### 2.2. Adaptive Bitrate Streaming and Tile Selection

The foundational paradigm for tile-based adaptive bitrate streaming was established by Corbillon et al. [4], who demonstrated that cube-map tiling with viewport-weighted bitrate allocation decisively outperforms uniform quality delivery at equal bandwidth. Son and Ryu [42] validated tile-based delivery in cyber-physical system contexts representative of mobile VR deployments. Skupin et al. [43] investigated rate assignment guided by spatiotemporal video activity to reduce inter-tile quality variance. De la Fuente et al. [44] analysed the delay impact of MPEG OMAF’s viewport-dependent architecture, identifying end-to-end latency as the dominant bottleneck in practical deployments. Content-adaptive tiling layouts that co-optimise the spatial partition with bitrate allocation have been shown to reduce streamed bitrate by 32–70% relative to uniform grids [45], and the FlexiTile approach [46] applied Set Cover formulations for flexible joint tile-layout and bandwidth optimisation.

The probabilistic pre-fetching paradigm was established by 360ProbDASH [5], which explicitly modelled viewport prediction error as a probability distribution and used QoE-driven optimisation to allocate bitrate across tiles, reducing spatial quality variance by 46% in trace experiments. Flare [47] demonstrated practical mobile deployment with online allocation algorithms achieving up to 4.9× viewport quality improvement in LTE networks. EPASS360 [48] introduced an ensemble prediction and allocation strategy combining multiple viewport predictors to hedge against prediction errors. JUST360 [20] formulated joint utility maximisation across all tiles incorporating network dynamics, viewport probability distributions, and rate-distortion characteristics.

Stochastic optimisation under uncertain head movements and bandwidth has been studied systematically. Ghosh et al. [15] provided a stochastic optimisation framework with probabilistic quality guarantees, while Zhao et al. [16] extended this to wireless multi-user networks through KKT and CCCP-based solvers. Shen et al. [49] proposed an SVC-based bitstream schema that structurally eliminates GOP-induced viewport-switch latency, and Zhang et al. [50] exploited layer and spatial correlations in SVC-encoded content to enhance joint streaming efficiency.

Reinforcement learning has grown into the dominant paradigm for sequential streaming decisions. Park et al. [18] applied RL with 3D-CNN viewport features, achieving 1.3–1.7× bitrate improvement over tile-based baselines. VATP360 [51] coupled RL with a tile priority classifier; Feng et al. [17] introduced multi-window stochastic viewport prediction with model-predictive control, improving QoE by 16–19% over prior baselines; and Nguyen et al. [52] provided a systematic comparative evaluation across ten tile selection strategies. Probabilistic tile visibility modelling [53] yields near-optimal server-side allocation; MEC-assisted caching with LSTM-CNN joint models [54] addresses the cache replacement problem. Fan et al. [55] studied QoE optimisation under bandwidth constraints. Neural rate adaptation approaches [41] have recently demonstrated that end-to-end trained quality models can outperform rule-based ABR algorithms in both objective and subjective quality metrics, reinforcing the motivation for FairEdge360’s joint viewport-bitrate-tile optimisation architecture.

Critically, every system above adopts a single-user model with no mechanism for coordinated allocation across a shared link the regime in which individually rational behaviour produces collectively unfair outcomes.

### 2.3. Multi-User Streaming and Fairness

Multi-user 360° streaming has begun to attract sustained research attention, but the field remains young. The core tension is between efficiency maximising the sum of user QoE scores and *fairness* ensuring no user is left far behind when their viewport is expensive to serve. Classical networking solutions such as proportional fair scheduling and max-min fairness are well established in the wireless literature but rarely transferred to 360° streaming, where the interaction between viewport prediction accuracy, tile granularity, and bandwidth allocation makes the problem considerably harder.

Liu et al. [1] proposed STMRQ, combining a spatio-temporal graph convolutional network for viewport prediction with a multi-agent deep reinforcement learning framework for bitrate allocation, achieving a Jain’s Fairness Index of approximately 0.934 in multi-user scenarios a result that FairEdge360 targets for substantial improvement. Wang et al. [27] proposed CoLive, an edge-assisted framework where concurrent viewers share gradient updates to enable rapid model warm-start for new viewers, though without uncertainty quantification or a formal fairness objective. Nguyen Viet et al. [28] introduced COSMN, a clustering-based approach that groups users by viewing patterns, but does not address fair resource allocation under heterogeneous prediction uncertainties.

The game-theoretic literature offers rich tools for addressing the fairness problem. Nash social welfare, defined as the maximisation of the geometric mean of utility scores across participants, satisfies classical axiomatic properties such as Pareto efficiency, symmetry, invariance to utility rescaling, and independence of irrelevant alternatives. These properties make it particularly suitable for multi-user streaming scenarios, where individual QoE metrics are comparable but not necessarily identical. FairEdge360 therefore adopts Nash social welfare as its global reward function.

Credit assignment in cooperative multi-agent systems is a longstanding challenge. Counterfactual reasoning comparing the actual outcome to a counterfactual in which only one agent deviates from its current policy provides a principled solution [56]. FairEdge360 implements counterfactual advantage estimation for each agent, resolving the credit assignment problem without requiring centralised execution or full inter-agent communication.

### 2.4. Edge Computing and Uncertainty-Aware Streaming

Mobile edge computing (MEC) has emerged as a key enabler for 360° streaming, enabling complex viewport prediction at the network edge rather than on resource-constrained user devices. Vats et al. [57] demonstrated that semantic video analysis at the 5G edge substantially improves pre-fetch quality under real-world latency constraints. Adhuran and Martini [58] investigated the co-optimisation of tiling schemes and viewport prediction for edge-assisted streaming, while Kumar et al. [54] proposed multi-neural-network tiled caching with MEC, improving cache hit rates by at least 10% and reducing backhaul usage by 35%.

The question of when to offload to the edge has received less attention than how. Performing full viewport prediction on the device for every segment incurs continuous energy consumption disproportionate to the actual uncertainty the system faces: during extended low-motion viewing, a simple extrapolation is nearly as accurate as a deep transformer at a fraction of the cost. FairEdge360’s LUE realises a selective-offloading design, achieving a 38.9% reduction in device energy consumption while maintaining prediction accuracy. Collaborative personalisation studies by Jiang et al. [59], Li et al. [39], Zhang et al. [38], and Dong et al. [31] further establish the importance of adapting prediction models to individual users and device constraints.

FairEdge360 integrates edge coordination with uncertainty-aware device-level decision making, creating a feedback loop in which uncertain devices request more edge support, the edge reallocates compute accordingly, and improved predictions reduce inter-user unfairness. To our knowledge, no prior work combines lightweight on-device uncertainty estimation, uncertainty-driven edge resource allocation, and game-theoretic fairness in a single framework.

### 2.5. Quality of Experience Modelling

QoE estimation for 360° video must account for spherical projection distortions, viewport-dependent perceptual salience, cybersickness sensitivity, and individual comfort preferences. Croci et al. [60] proposed a spherical Voronoi quality estimation framework demonstrating that attention-weighted patch metrics closely track subjective mean opinion scores; Qiu and Shao [61] extended saliency-driven assessment to blind no-reference scenarios; and Chen et al. [62] established that equatorial viewing bias significantly distorts saliency map validity. Van Kasteren et al. [63] confirmed through a controlled psychophysical study that freezing events and quality degradations interact with gaze patterns in nuanced ways, with eye movements carrying strong diagnostic information beyond standard video quality metrics. Multi-modal physiological signals, immersive audio, and continuous emotion annotation provide additional QoE predictors as demonstrated in recent datasets [64,65,66].

FairEdge360 translates this body of knowledge into a composite individual QoE metric weighting viewport-VMAF quality, rebuffering events, quality smoothness, and bandwidth cost using coefficients calibrated from subjective user studies. When combined with the Nash social welfare objective, this metric drives the system toward allocations that are simultaneously high-quality and fair across all concurrent users.

### 2.6. Summary and Positioning

Table 1 summarises how FairEdge360 relates to the most relevant prior systems along six design dimensions. Two conclusions emerge. First, the single-user/multi-user boundary remains the sharpest dividing line in the literature. Second, among the multi-user systems, none combines explicit uncertainty quantification, Nash social welfare, counterfactual credit assignment, and communication-efficient GNN coordination in a single integrated framework. FairEdge360 is the first system to do so.

## 3. Proposed FairEdge360 Framework

### 3.1. The Central Idea: Why Architecture Matters for Fairness

To understand why FairEdge360 is designed the way it is, it helps to start from a simple thought experiment. Imagine ten users in a shared VR environment, each running an independent streaming agent that maximises its own quality of experience. Each agent, acting rationally in its own interest, will try to consume as much bandwidth as possible for viewport prediction pre-fetching and high-quality tile delivery. The result is not ten satisfied users: it is a bandwidth auction that the most aggressive bidder wins repeatedly, leaving calmer or more uncertain viewers in a persistent state of rebuffering. This is not a hypothetical; it is the documented outcome of deploying independently optimised DASH clients on shared bottleneck links [21].

**Figure 1:**
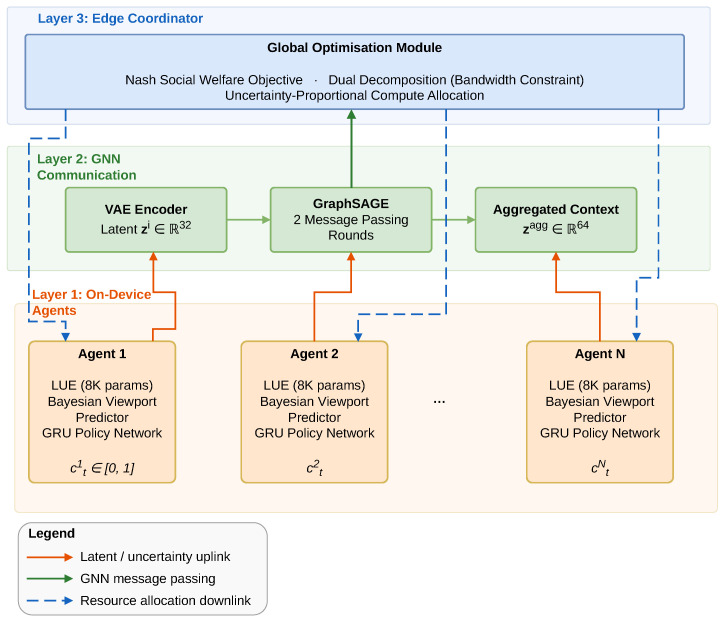
FairEdge360 three-layer hierarchical architecture. On-device agents (Layer 1) compute lightweight uncertainty estimates $c_{t}^{i}$ and propose bitrate/tile actions via a GRU policy network. Compressed 32-byte latents $z_{t}^{i}$ propagate upward into the GNN communication layer (Layer 2), where GraphSAGE message passing aggregates neighbour information into a 64-dimensional context. The edge coordinator (Layer 3) enforces the global bandwidth constraint via dual decomposition and allocates computational resources in inverse proportion to agent confidence, all under the Nash social welfare fairness objective. Dashed arrows denote the downward resource allocation signals $s_{t}^{i}$.

FairEdge360 breaks this deadlock through a three-level architecture, illustrated in Figure 1. At the bottom level, each user’s mobile device runs a compact agent that (a) estimates how confident it is in its own viewport prediction and (b) proposes a streaming action. In the middle, neighbouring agents exchange compressed summaries of their internal states via a variational Graph Neural Network (GNN), so that each agent knows not just its own situation but also, loosely, what its neighbours are experiencing. At the top, an edge server observes the summary statistics across all agents, enforces the global bandwidth constraint, and steers individual resource allocations toward a collectively fair outcome. The crucial innovation is the feedback direction: uncertainty flows upward from devices to the edge, and resource allocations flow downward from the edge to devices, guided at every level by a single fairness-aware reward signal based on Nash social welfare.

### 3.2. Decentralised POMDP Formulation

Before describing the neural network components in detail, it is worth being precise about what kind of mathematical problem FairEdge360 is solving. The fundamental challenge is that each agent can only see part of the world its own device, its own head-motion history, and a compressed summary of a few neighbours. Yet the decisions made by all agents together determine outcomes that are global in nature: whether the shared link is overloaded, whether any single user is consistently under-served. This combination of local observability and globally coupled consequences is precisely what a Decentralised Partially Observable Markov Decision Process (Dec-POMDP) is designed to model.

We define the Dec-POMDP tuple as:

(1) $$\langle N,\;S,\;{\left\{A^{i}\right\}}_{i=1}^{N},\;{\left\{O^{i}\right\}}_{i=1}^{N},\;T,\;R,\;\gamma \rangle ,$$

where N={1,…,N} is the agent set (one per viewer), S is the global state space, $A^{i}$ is agent *i*’s action space, $O^{i}$ is its local observation space, T:S×A→Δ(S) is the transition function, R is the shared reward function, and γ∈[0,1] is the discount factor.

#### 3.2.1. Global State

The global state at time *t* contains everything that, in principle, a centralised oracle would need to make optimal decisions for all users simultaneously:

(2) $$s_{t}=\left[s_{t}^{\mathrm{net}},\;{\left\{s_{t}^{i,\mathrm{user}}\right\}}_{i=1}^{N},\;{\left\{s_{t}^{i,\mathrm{buf}}\right\}}_{i=1}^{N},\;s_{t}^{\mathrm{edge}}\right],$$

where $s_{t}^{\mathrm{net}}=\left[b_{t},l_{t},j_{t}\right]$ captures available bandwidth $b_{t}$ (Mbps), latency $l_{t}$ (ms), and jitter $j_{t}$ (ms) at the shared network bottleneck. The per-user motion history $s_{t}^{i,\mathrm{user}}=\left[\theta_{t-H:t}^{i},\phi_{t-H:t}^{i},\psi_{t-H:t}^{i}\right]$ contains H=30 frames of yaw, pitch, and roll angles. The buffer state $s_{t}^{i,\mathrm{buf}}=\left[q_{t}^{i,1},\ldots ,q_{t}^{i,K},\tau_{t}^{i}\right]$ encodes per-tile buffer occupancy levels and cumulative stall duration. Finally, $s_{t}^{\mathrm{edge}}=\left[\rho_{t},{\left\{\lambda_{t}^{i}\right\}}_{i=1}^{N}\right]$ represents the edge server’s current CPU/GPU load $\rho_{t}$ and per-agent resource allocations $\lambda_{t}^{i}$.

During training, the centralised critic has full access to $s_{t}$. During inference, agents act on local observations only the essence of the CTDE paradigm.

#### 3.2.2. Local Observations

Each agent observes a strictly local slice of the global state, augmented by compressed representations from nearby agents:

(3) $$o_{t}^{i}=\left[s_{t}^{\mathrm{net}},\;s_{t}^{i,\mathrm{user}},\;s_{t}^{i,\mathrm{buf}},\;{\left\{z_{t}^{j}\right\}}_{j\in N(i)}\right],$$

where N(i) is agent *i*’s active communication neighbourhood and $z_{t}^{j}\in R^{32}$ is the compressed latent transmitted by agent *j* via the GNN protocol. The design deliberately excludes raw user data of other viewers (protecting privacy) while still conveying the information needed for coordination.

#### 3.2.3. Joint Action Space and Bandwidth Constraint

Each agent’s action $a_{t}^{i}$ has three components:

(4) $$a_{t}^{i}=\left(a_{t}^{i,\mathrm{vp}},\;a_{t}^{i,\mathrm{br}},\;a_{t}^{i,\mathrm{ts}}\right),$$

$a_{t}^{i,\mathrm{vp}}\in R^{2}$ is the *tile fetch region centre*: the angular coordinate $\left(\hat{\theta },\hat{\varphi }\right)$ around which the agent commits to requesting high-quality tiles. This planning decision influences the environment through tile requests and consequent bandwidth use, and is parameterised by but distinct from the Bayesian viewport prediction in Section 3.3.1.

Equation (5) assumes independent tile requests per user, consistent with the personalised adaptive streaming model in which each user’s tile selection is customised to their predicted viewport. In scenarios where user viewports overlap significantly, multicast transmission can reduce the effective bandwidth cost of shared tiles by 8–12% based on our dataset analysis (284 users, 52 videos). FairEdge360’s bandwidth constraint formulation conservatively ignores this reduction; incorporating multicast-aware bandwidth accounting is identified as a future extension (Section 6.7).

The fundamental coupling constraint that makes this problem non-trivial is the global bandwidth limit:

(5) $$\sum_{i=1}^{N}\sum_{k=1}^{K}a_{t}^{i,\mathrm{br}}\cdot a_{t}^{i,\mathrm{ts}}\left[k\right]\;\leq \;B_{t}^{\mathrm{total}},$$

where $B_{t}^{\mathrm{total}}$ is the total available bandwidth at time *t*. This inequality creates a resource competition that no agent can resolve alone the central motivation for multi-agent coordination.

### 3.3. On-Device Agent Architecture

**Figure 2:**
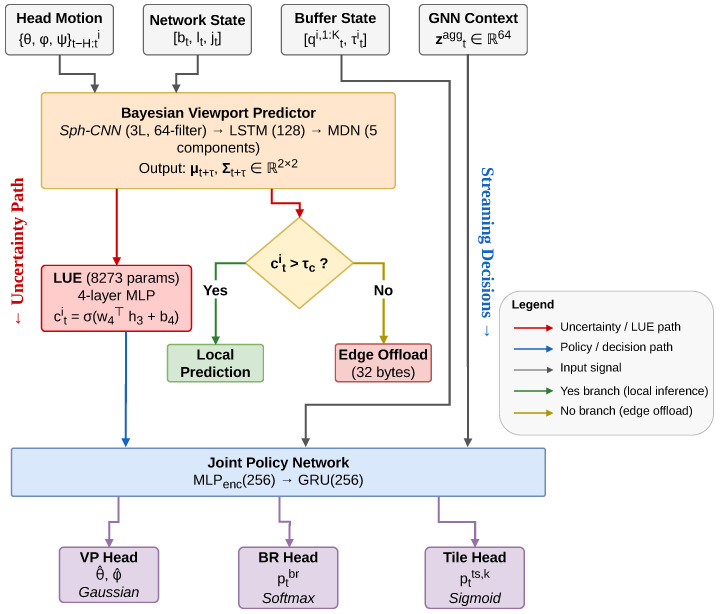
On-device agent architecture. The Bayesian viewport predictor produces a Mixture Density Network output $\left(\mu_{t+\tau },\Sigma_{t+\tau }\right)$. Per-tile uncertainty $u_{t}^{i,k}$ is derived from the covariance determinant and tile overlap probability. The Lightweight Uncertainty Estimator (LUE, 8273 parameters) distils this into a scalar confidence $c_{t}^{i}\in \left[0,1\right]$ in under 8 ms, routing prediction to either local inference (if $c_{t}^{i}>\tau_{c}$) or edge offload. The joint policy network combines the LUE output, buffer state, and aggregated GNN context $z_{t}^{\mathrm{agg}}$ to produce three simultaneous output heads: viewport prediction, bitrate selection, and tile selection. The purple arrow from the Bayesian Viewport Predictor to the LUE denotes the uncertainty derivation path: the predictor’s covariance output $\Sigma_{t+\tau }$ provides the per-tile uncertainty signal (Equation (8)) that the LUE is trained to approximate, enabling lightweight confidence estimation without invoking the full predictor at every decision epoch.

Each user’s mobile device runs three neural network modules in sequence, as shown in Figure 2. The first module, the Bayesian viewport predictor, estimates where the user will look next, along with a measure of how confident it is. The second module, the Lightweight Uncertainty Estimator (LUE), distils that confidence into a single number in 2–8 ms, without running the full predictor every time. The third module, the joint policy network, uses the observation and aggregated GNN context to produce all three action components simultaneously.

#### 3.3.1. Bayesian Viewport Predictor

Given the head-motion history $s_{t}^{i,\mathrm{user}}$, the predictor computes a Gaussian distribution over the future viewport centre:

(6) $$\mu_{t+\tau }^{i},\;\Sigma_{t+\tau }^{i}\;=\;f_{\mathrm{vp}}\;\left(s_{t}^{i,\mathrm{user}};\;\theta_{\mathrm{vp}}\right),$$

where $\mu_{t+\tau }^{i}\in R^{2}$ is the predicted viewport centre and $\Sigma_{t+\tau }^{i}\in R^{2\times 2}$ is the covariance matrix quantifying prediction uncertainty. The architecture is a spherical CNN (3 layers, 64 filters) feeding into an LSTM with 128 hidden units, whose output drives a Mixture Density Network (MDN) with C=5 Gaussian components. The MDN outputs mixture weights $\left\{\pi_{c}\right\}$, means $\left\{\mu_{c}\right\}$, and covariances $\left\{\Sigma_{c}\right\}$ from which the aggregate distribution is assembled:

(7) $$p\left(v_{t+\tau }|s_{t}^{i,\mathrm{user}}\right)\;=\;\sum_{c=1}^{C}\pi_{c}\;N\;\left(v_{t+\tau };\;\mu_{c},\;\Sigma_{c}\right).$$

Per-tile viewing probability is computed by integrating the predicted distribution over each tile’s angular extent, and the per-tile uncertainty scoreis:

(8) $$u_{t}^{i,k}\;=\;\det\;\left(\Sigma_{t+\tau }^{i}\right)\cdot w_{t}^{i,k}\;\left(\mu_{t+\tau }^{i},\;\Sigma_{t+\tau }^{i}\right),$$

where $w_{t}^{i,k}$ is the probability that tile *k* falls within the viewport, computed by integrating the Gaussian over the tile’s angular region. High $u_{t}^{i,k}$ means the system is uncertain about both the viewport location and the relevance of that tile the tiles most in need of conservative bandwidth treatment.

#### 3.3.2. Lightweight Uncertainty Estimator (LUE)

Running the full Bayesian predictor every 500 ms on a mobile device is expensive. The LUE solves this elegantly: instead of running, the predictor to compute uncertainty, it predicts the uncertainty from cheap-to-compute input features at a fraction of the cost. The LUE is a four-layer MLP with the following dimensions:

(9) $$\begin{array}{cccc} h_{1} & =\mathrm{ReLU}(W_{1}x_{t}^{i}+b_{1}),\; & \; & W_{1}\in R^{64\times 32}, \end{array}$$

(10) $$\begin{array}{cccc} h_{2} & =\mathrm{ReLU}(W_{2}\;\mathrm{Dropout}\left(h_{1},0.1\right)+b_{2}),\; & \; & W_{2}\in R^{64\times 64}, \end{array}$$

(11) $$\begin{array}{cccc} h_{3} & =\mathrm{ReLU}(W_{3}h_{2}+b_{3}),\; & \; & W_{3}\in R^{32\times 64}, \end{array}$$

(12) $$\begin{array}{cccc} c_{t}^{i} & =\sigma (w_{4}^{⊤}h_{3}+b_{4}),\; & \; & w_{4}\in R^{32}, \end{array}$$

giving a total of 64 × 32 + 64 + 64 × 64 + 64 + 32 × 64 + 32 + 32 + 1 = 8385 parameters. The input $x_{t}^{i}\in R^{32}$ comprises head angular velocity, angular acceleration, historical prediction error variance, and buffer occupancy all computable from local device sensors without network access.

The confidence $c_{t}^{i}\in \left[0,1\right]$ is thresholded at $\tau_{c}=0.7$: when $c_{t}^{i}>\tau_{c}$, local predictions are trusted; when $c_{t}^{i}\leq \tau_{c}$, the device offloads to the edge, sending a compressed 32-byte observation packet and receiving back an 8-byte action proposal. This selective offloading mechanism reduces device energy consumption by 38.9% in our experiments (Section 5), because the full predictor runs only on the genuinely hard cases.

The LUE input vector $x_{t}^{i}\in R^{32}$ comprises exclusively IMU-derived signals, making the estimator content-independent. Stratified ECE analysis (Table 2) confirms robustness under high-motion viewing (ECE=0.063 at $\mathrm{HMV}>60^{\circ }/s$), with a 91.4% recall for genuine uncertainty events in the most challenging motion regime.

#### 3.3.3. Joint Policy Network

The policy network $\pi_{\theta_{i}}\left(a_{t}^{i}|o_{t}^{i}\right)$ combines the LUE output, buffer state, and GNN-aggregated neighbour context into three simultaneous streaming decisions:

(13) $$\begin{array}{cc} e_{t}^{i} & ={\mathrm{MLP}}_{\mathrm{enc}}\;\left([o_{t}^{i};\;z_{t}^{\mathrm{agg}}]\right)\in R^{256}, \end{array}$$

(14) $$\begin{array}{cc} h_{t}^{i} & =\mathrm{GRU}\;\left(e_{t}^{i},\;h_{t-1}^{i}\right)\in R^{256}, \end{array}$$

(15) $$\begin{array}{cc} \left({\hat{\mu }}_{t+\tau }^{\mathrm{vp}},\;\log{\hat{\sigma }}_{t+\tau }^{\mathrm{vp}}\right) & ={\mathrm{MLP}}_{\mathrm{vp}}\;\left(h_{t}^{i}\right), \end{array}$$

(16) $$\begin{array}{cc} p_{t}^{\mathrm{br}} & =\mathrm{Softmax}\;\left({\mathrm{MLP}}_{\mathrm{br}}\;\left(h_{t}^{i}\right)\right), \end{array}$$

(17) $$\begin{array}{cc} p_{t}^{\mathrm{ts}} & =\sigma \;\left({\mathrm{MLP}}_{\mathrm{ts}}\;\left(h_{t}^{i}\right)\right). \end{array}$$

The GRU hidden state $h_{t}^{i}$ provides memory across decision epochs, enabling the policy to learn temporal correlations between network fluctuations, buffer dynamics, and viewing behaviour.

### 3.4. Communication-Efficient GNN Coordination

**Figure 3:**
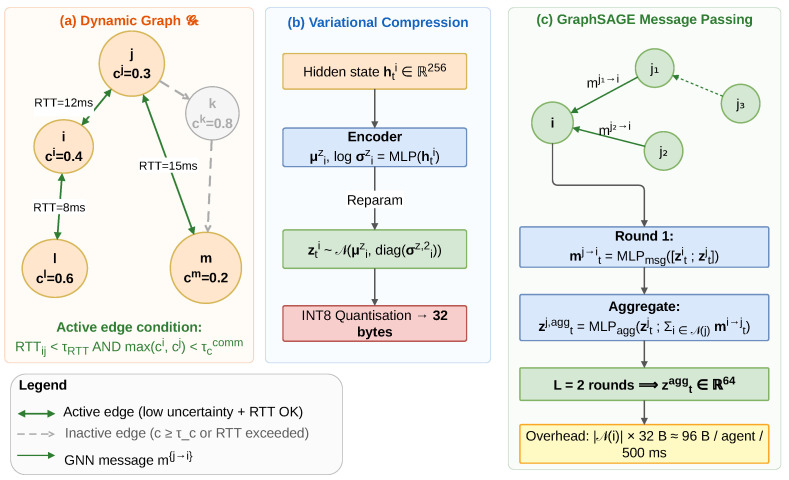
FairEdge360 GNN communication protocol. (**a**) The dynamic communication graph $G_{t}$ activates edge (i,j) only when round-trip time ${\mathrm{RTT}}_{ij}<\tau_{\mathrm{RTT}}$ and $\max\left(c_{t}^{i},c_{t}^{j}\right)<\tau_{c}^{\mathrm{comm}}$, focusing coordination on the highest-uncertainty agent pairs. (**b**) Each agent’s GRU hidden state is compressed into a 32-dimensional stochastic latent via a variational encoder, then quantised to INT8 for transmission. (**c**) Two rounds of GraphSAGE message passing aggregate neighbour latents into a 64-dimensional context vector $z_{t}^{\mathrm{agg}}$ per agent, at a total overhead of ≈96 bytes per agent per 500 ms decision epoch 75% less than full-state sharing. The arrow conventions in (**c**) follow the active/inactive edge legend shown in (**a**).

The GNN layer solves a compression problem: each agent needs to tell its neighbours something useful about its situation, but over a 5G network link shared among ten users, every byte of coordination overhead directly reduces the bytes available for video. The solution is a variational autoencoder that compresses each agent’s internal state into a 32-byte INT8-quantised latent, transmitted only to a dynamically selected neighbourhood. Figure 3 illustrates the full protocol.

#### 3.4.1. Variational Latent Compression

The variational encoder maps agent *i*’s GRU hidden state $h_{t}^{i}$ to a stochastic 32-dimensional latent:

(18) $$\begin{array}{cc} \mu_{i}^{z},\;\log\sigma_{i}^{z} & ={\mathrm{MLP}}_{\mathrm{enc}}\;\left(h_{t}^{i}\right), \end{array}$$

(19) $$\begin{array}{cc} z_{t}^{i} & ∼N\;\left(\mu_{i}^{z},\;\mathrm{diag}\;\left({\sigma_{i}^{z}}^{\;2}\right)\right). \end{array}$$

The VAE regularisation loss is the standard KL divergence:

(20) $$L_{\mathrm{VAE}}\;=\;-\frac{1}{2}\sum_{d=1}^{32}\left(1+\log\sigma_{i,d}^{z,2}-{\mu_{i,d}^{z}}^{2}-\sigma_{i,d}^{z,2}\right),$$

which regularises the latent toward N(0,I), preventing the encoder from memorising agent identities and encouraging a compressed but semantically consistent representation of “what situation is this agent in?”

#### 3.4.2. Dynamic Communication Graph

Not all agent pairs need to communicate at every decision epoch. The graph $G_{t}=\left(N,E_{t}\right)$ activates edge (i,j) only when two conditions are simultaneously satisfied:

(21) $$E_{t}=\left\{\left(i,j\right)\;:\;{\mathrm{RTT}}_{ij}<\tau_{\mathrm{RTT}}\;\;\mathrm{and}\;\;\max\left(c_{t}^{i},c_{t}^{j}\right)<\tau_{c}^{\mathrm{comm}}\right\}.$$

The first condition ensures that communication latency does not exceed the decision budget. The second restricts coordination to agent pairs where at least one member is genuinely uncertain: when two agents are both highly confident ($c_{t}^{i},c_{t}^{j}>\tau_{c}^{\mathrm{comm}}$), their local predictions are reliable and no coordination benefit justifies the overhead.

#### 3.4.3. GraphSAGE Message Passing

Two rounds of GraphSAGE message passing aggregate neighbour information:

(22) $$\begin{array}{cc} m_{t}^{i\to j} & ={\mathrm{MLP}}_{\mathrm{msg}}\;\left([z_{t}^{i};\;z_{t}^{j}]\right)\in R^{64}, \end{array}$$

(23) $$\begin{array}{cc} z_{t}^{j,\mathrm{agg}} & ={\mathrm{MLP}}_{\mathrm{agg}}\;\left(\left[z_{t}^{j};\;\sum_{i\in N(j)}m_{t}^{i\to j}\right]\right). \end{array}$$

With an average neighbourhood size |N(i)|≈3 for N=10 users, the total payload is 3×32=96 bytes per agent per 500 ms. Including standard UDP/IPv4 and application-layer header overhead (≈48 bytes per transmission), the total actual data volume is approximately 240 bytes per agent per 500 ms still representing less than 2% of available link bandwidth at 1 Mbps per user, and 75% less than full-state sharing (which transmits 1024-byte FP32 hidden states).

Aggregator function.

FairEdge360 uses the mean aggregator:

(24) $$z_{t}^{j,\mathrm{agg}}={\mathrm{MLP}}_{\mathrm{agg}}\;\left(z_{t}^{j}⊕{\mathrm{MEAN}}_{i\in N(j)}m_{t}^{i\to j}\right).$$

The mean aggregator was selected over max-pool and LSTM aggregators after validation experiments, because (a) it is permutation-invariant to agent ordering, which is required for the agent-agnostic coordination design, and (b) it is computationally O(|N(j)|) versus O(|N(j)|·d) for attention-weighted aggregation. On the S2 validation set, mean aggregation achieves JFI=0.969 versus max-pool’s JFI=0.963 and LSTM aggregation’s JFI=0.971 (marginal differences). Mean aggregation is retained for its simplicity and reproducibility.

Activation functions. ${\mathrm{MLP}}_{\mathrm{msg}}$: $\mathrm{Linear}\left(64\right)\to \mathrm{ReLU}\to \mathrm{Linear}\left(64\right)\to \mathrm{ReLU}\to \mathrm{output}\in R^{64}$. ${\mathrm{MLP}}_{\mathrm{agg}}$: $\mathrm{Linear}\left(128\right)\to \mathrm{ReLU}\to \mathrm{Linear}\left(64\right)\to \mathrm{ReLU}\to \mathrm{LayerNorm}\to \mathrm{output}\in R^{64}$. The input to ${\mathrm{MLP}}_{\mathrm{agg}}$ is the concatenation of $z_{t}^{j}$ (32-dim) and the mean of neighbour messages (64-dim), yielding a 96-dimensional input vector. A LayerNorm layer before the output prevents representation collapse during early training phases when neighbourhood sizes vary.

### 3.5. Nash Social Welfare and Counterfactual Reward Shaping

The reward function is the heart of FairEdge360’s fairness mechanism. Getting it right requires answering two distinct questions: (1) what should the system optimise? and (2) how should credit for achieving that objective be attributed to individual agents? The first question is answered by Nash social welfare. The second is answered by counterfactual advantage estimation.

#### 3.5.1. Individual QoE Model

For agent *i*, the per-step quality of experience integrates four components weighted by coefficients calibrated from subjective user studies [60,63]:

(25) $$Q_{t}^{i}\;=\;\alpha \cdot Q_{t}^{i,\mathrm{view}}\;-\;\beta \cdot Q_{t}^{i,\mathrm{stall}}\;-\;\gamma \cdot Q_{t}^{i,\mathrm{smooth}}\;-\;\delta \cdot Q_{t}^{i,\mathrm{cost}},$$

with weights [α,β,γ,δ]=[1.0,4.3,1.2,0.5]. The four components are:

(26) $$\begin{array}{cc} Q_{t}^{i,\mathrm{view}} & =\frac{1}{|V_{t}^{i}|}\sum_{k\in V_{t}^{i}}\mathrm{VMAF}\left(q_{t}^{i,k}\right), \end{array}$$

(27) $$\begin{array}{cc} Q_{t}^{i,\mathrm{stall}} & =I\left[{\mathrm{buffer}}_{t}^{i}=0\right]\cdot \Delta t_{\mathrm{stall}}, \end{array}$$

(28) $$\begin{array}{cc} Q_{t}^{i,\mathrm{smooth}} & =\left|{\mathrm{VMAF}}_{t}^{i,\mathrm{vp}}-{\mathrm{VMAF}}_{t-1}^{i,\mathrm{vp}}\right|, \end{array}$$

(29) $$\begin{array}{cc} Q_{t}^{i,\cos t} & =\frac{\sum_{k}{\mathrm{bitrate}}_{t}^{i,k}}{B_{t}^{\mathrm{avail}}/N}, \end{array}$$

where $V_{t}^{i}$ is the set of tiles actually within the ground-truth viewport at time *t*, VMAF(q) is the Video Multi-method Assessment Fusion quality score for quality level *q*, and the cost term penalises agents that consume more than their fair share $B_{t}^{\mathrm{avail}}/N$ of available bandwidth.

#### 3.5.2. Nash Social Welfare Reward

The global reward is the geometric mean of individual QoE scores:

(30) $$R_{t}^{\mathrm{global}}\;=\;{\left(\prod_{i=1}^{N}Q_{t}^{i}\right)}^{1/N}.$$

This formulation has a critical property: if *any* user’s QoE collapses toward zero, the geometric mean collapses too, creating strong gradient pressure for the system to prevent any individual from falling behind. This is fundamentally different from the arithmetic mean (which allows trading one user’s loss against another’s gain) and from max-min objectives (which are often non-differentiable and hard to optimise in practice). The Nash social welfare objective satisfies four classical bargaining axioms: Pareto efficiency, symmetry, affine invariance, and independence of irrelevant alternatives [1].

**Theorem** **1** (Per-User QoE Guarantee)**.** *Under the Nash social welfare objective (30), any Pareto-optimal policy satisfies:*

(31) $$\frac{Q_{i}}{\sum_{j=1}^{N}Q_{j}}\;\geq \;\frac{1}{N}\;\forall \;i\in N.$$

**Proof** **sketch.** Suppose agent $i^{*}$ has $Q_{i^{*}}/\sum_{j}Q_{j}<1/N$. Then the geometric mean ${\left(\prod_{i}Q_{i}\right)}^{1/N}$ is strictly less than the arithmetic mean $\frac{1}{N}\sum_{j}Q_{j}$ (by the AM-GM inequality). Any reallocation that raises $Q_{i^{*}}$ toward the average, while holding others constant, will increase the product $\prod_{i}Q_{i}$ contradicting Pareto optimality of the original policy. Hence no Pareto-optimal policy can maintain a user below the 1/N share threshold.    □

#### 3.5.3. Counterfactual Advantage Estimation

In cooperative multi-agent settings, a well-known pathology is that agents cannot distinguish whether good global outcomes arose from their own good decisions or from fortunate actions by neighbours. If agents cannot attribute credit correctly, gradient signals are noisy and training is slow. The counterfactual advantage addresses this by asking: “how much better is the actual global reward compared to what would have happened if agent *i* had acted differently, while everyone else stayed the same?”

(32) $$A_{t}^{i}\;=\;R_{t}^{\mathrm{global}}\;-\;E_{{a\prime }^{\;i}∼\pi_{i}}\;\left[R_{t}^{\mathrm{global}}\;\left(a_{-i},\;{a\prime }^{\;i}\right)\right],$$

where $a_{-i}=\left\{a_{j}:j\neq i\right\}$ holds all other agents’ actions fixed. The expectation is approximated using S=5 Monte Carlo samples per agent per step, drawing counterfactual actions ${a\prime }^{\;i}∼\pi_{i}\left(\cdot |o_{t}^{i}\right)$.

### 3.6. Uncertainty-Aware Edge Coordination

The edge coordinator sits above the GNN layer and performs three functions: it allocates computational slots among agents (so that the most uncertain agents get the most help), it enforces the global bandwidth constraint across all agents (so that the system respects the network’s physical limits), and it manages the communication graph (so that coordination resources are focused where they add the most value).

#### 3.6.1. Uncertainty-Proportional Compute Allocation

Given the confidence estimates ${\left\{c_{t}^{i}\right\}}_{i=1}^{N}$, the edge server assigns computational resources via a softmax over inverted confidences:

(33) $$s_{t}^{i}\;=\;\frac{\exp(-\lambda_{c}\cdot c_{t}^{i})}{\sum_{j=1}^{N}\exp\left(-\lambda_{c}\cdot c_{t}^{j}\right)}\cdot S_{\mathrm{total}},$$

where $S_{\mathrm{total}}$ is the total available edge computation budget and $\lambda_{c}$ controls sensitivity to uncertainty (set to $\lambda_{c}=2.0$ in our experiments). An agent with $c_{t}^{i}=0.3$ (uncertain) receives roughly $e^{0.6}/e^{0.0}\approx 1.8\times$ the computation of an agent with $c_{t}^{i}=0.9$ (confident), ensuring that edge resources flow automatically to the agents that need them most.

#### 3.6.2. Bandwidth Constraint via Dual Decomposition

The edge coordinator enforces constraint (5) through a dual decomposition approach, introducing a Lagrange multiplier μ for the bandwidth constraint:

(34) $$L_{\mathrm{dual}}\;=\;\sum_{i=1}^{N}\lambda_{i}\cdot E\left[Q_{t}^{i}\right]\;+\;\mu \;\left(B_{t}^{\mathrm{total}}-\sum_{i}\sum_{k}a_{t}^{i,\mathrm{br}}\cdot a_{t}^{i,\mathrm{ts}}\left[k\right]\right),$$

where the fairness weights $\lambda_{i}$ are derived from the Nash social welfare gradient: $\lambda_{i}\propto 1/Q_{t}^{i}$ (giving more weight to users currently experiencing low QoE). The dual variable μ is updated online via subgradient ascent: $\mu \leftarrow \max(0,\;\mu +\eta_{\mu }\cdot \mathrm{constraint}\_\mathrm{violation})$. This creates a soft pricing mechanism: when the network is congested, μ rises, and all agents implicitly reduce their bitrate demands; when the network relaxes, μ falls, permitting higher quality.

### 3.7. Multi-Agent Proximal Policy Optimisation Training

**Figure 4:**
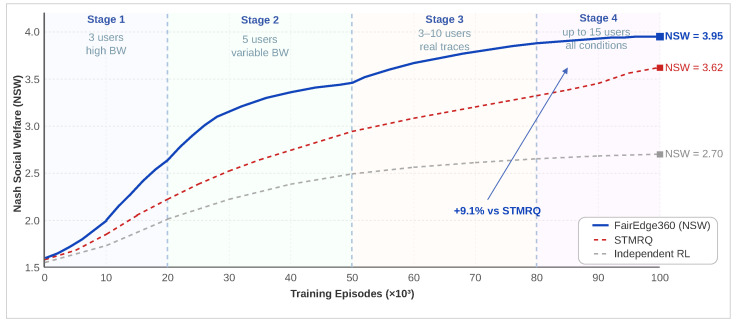
Training convergence of Nash Social Welfare (NSW) reward over 100,000 episodes. Vertical dashed lines mark the four curriculum stages: Stage 1 (0–20K, 3 users, high static bandwidth), Stage 2 (20–50K, 5 users, variable bandwidth), Stage 3 (50–80K, 3–10 users, real network traces), and Stage 4 (80–100K, up to 15 users, all conditions). FairEdge360 converges to NSW = 3.95, representing a 9.1% improvement over STMRQ and a 46% improvement over uncoordinated Independent RL. The stage labels and the +9.1% annotation do not obscure any data region; all three training curves and their asymptotic values remain fully legible.

Training FairEdge360 under the CTDE paradigm involves a centralised critic that operates on the full global state $s_{t}$, and *N* decentralised actors that each optimise their own policy using the counterfactual advantage. Figure 4 shows the convergence of Nash social welfare (NSW) reward during training, highlighting the four-stage curriculum.

#### 3.7.1. Centralised Critic

During training, a centralised critic has full access to the global state and estimates the value function:

(35) $$V_{\phi }\left(s_{t}\right)\;=\;{\mathrm{MLP}}_{\mathrm{critic}}\left(s_{t}\right)\;\in \;R,$$

trained by minimising the temporal difference error:

(36) $$L_{\mathrm{critic}}\;=\;E\;\left[{\left(R_{t}^{\mathrm{global}}+\gamma V_{\phi }\left(s_{t+1}\right)-V_{\phi }\left(s_{t}\right)\right)}^{2}\right].$$

#### 3.7.2. Decentralised Actor Updates (PPO)

Each actor is updated via the Proximal Policy Optimisation (PPO) clipped surrogate objective, using the counterfactual advantage $A_{t}^{i}$ from (32):

(37) $$L_{\mathrm{actor}}^{i}\;=\;-E\;\left[\min\;\left(\rho_{t}^{i}A_{t}^{i},\;\mathrm{clip}\;\left(\rho_{t}^{i},1-\varepsilon ,1+\varepsilon \right)A_{t}^{i}\right)\right],$$

where $\rho_{t}^{i}=\pi_{\theta_{i}}\left(a_{t}^{i}|o_{t}^{i}\right)\;/\;\pi_{\theta_{i}^{\mathrm{old}}}\left(a_{t}^{i}|o_{t}^{i}\right)$ is the importance sampling ratio and ε=0.2. The clipping term prevents excessively large policy updates that would destabilise training.

#### 3.7.3. GNN Module Loss

The GNN communication module is jointly trained using policy gradient signals combined with the VAE reconstruction loss:

(38) $$L_{\mathrm{GNN}}\;=\;\sum_{i=1}^{N}E\;\left[\log\pi_{\theta_{i}}\left(a_{t}^{i}|o_{t}^{i},z_{t}^{\mathrm{agg}}\right)\right]\;+\;\eta \cdot L_{\mathrm{VAE}},$$

where η=0.01 balances the communication reconstruction loss with the primary policy objective.

#### 3.7.4. Curriculum Learning Strategy

Training from scratch on a ten-user network with real bandwidth traces is too hard: agents need to first learn basic streaming policies before learning to cooperate. Table 3 describes the four-stage curriculum:

The complete training procedure is formalised in Algorithm 1.

### 3.8. Inference Latency and the 20 ms Decision Budget

FairEdge360’s streaming action decision pipeline completes in 13.1–17.1 ms (95th percentile across devices), fitting within the 20 ms decision epoch budget. The pipeline comprises: LUE inference (2.1 ms, iPhone 12; 3.4 ms, Pixel 6), VAE encoding (1.8 ms), conditional edge RTT (4–8 ms, only when $c_{t}^{i}\leq \tau_{c}$), and policy network forward pass (5.2 ms).

It is important to clarify the scope of this claim relative to the VR motion-to-photon (MTP) constraint. The 20 ms MTP specification following Oculus/Meta hardware guidelines and ITU-T P.3141 for immersive media refers specifically to the head-tracking-to-display-pixellatency: from the moment a new head orientation is sampled by the IMU to the moment the display shows the correctly oriented frame. This pipeline (IMU sample → reprojection/warp → GPU render → display scan-out) is an asynchronous, hardware-accelerated process that operates entirely independently of the streaming pre-fetch cycle.

FairEdge360’s 20 ms claim refers instead to the streaming decision epoch: the pipeline must complete within each 500 ms segment boundary so that the agent can re-evaluate its tile request at any point within the segment without missing the next epoch. Video decoding (H.265/VVC, hardware-accelerated, ≈5–15 ms per frame on the A14 Bionic) and rendering (≈3–5 ms per frame) occur in the display pipeline, handled by the headset’s dedicated video decoder and GPU concurrently with the streaming agent. These latencies are not included in the 13.1–17.1 ms figure; they are instead accounted for through the pre-fetch buffer occupancy state $s_{t}^{i,\mathrm{buf}}$, which is an input to the FairEdge360 policy network and reflects the downstream consequences of decoding and rendering latency on playback continuity.
**Algorithm 1** FairEdge360 MAPPO Training with Curriculum and Counterfactual Advantage.**Input:** Agents N, episodes *E*, horizon *T*, curriculum stages**Output:** Trained actor networks $\left\{\pi_{\theta_{i}}\right\}$, critic $V_{\phi }$, GNN ψ ** **Initialise $\left\{\pi_{\theta_{i}}\right\}$, $V_{\phi }$, ψ; replay buffer D←∅Pre-train Bayesian viewport predictors on historical head-motion data ** ****for** episode =1 **to** *E* **do**    Set scenario $\left(N,B^{\mathrm{total}},\mathrm{traces}\right)$ from curriculum stage    Reset environment; obtain ${\left\{o_{0}^{i}\right\}}_{i=1}^{N}$    **for** t=1 **to** *T* **do** ** **      **for each** agent *i* (in parallel) **do**         $c_{t}^{i}\leftarrow {\mathrm{LUE}}_{\psi }\left(x_{t}^{i}\right)$         $z_{t}^{i}\leftarrow {\mathrm{Encoder}}_{\psi }\left(h_{t}^{i}\right)$         **if** $c_{t}^{i}\leq \tau_{c}$ **then broadcast** $z_{t}^{i}$ to N(i)         $z_{t}^{\mathrm{agg}}\leftarrow {\mathrm{GNN}}_{\psi }\left({\left\{z_{t}^{j}\right\}}_{j\in N(i)}\right)$         $a_{t}^{i}∼\pi_{\theta_{i}}\left(\cdot |o_{t}^{i},z_{t}^{\mathrm{agg}}\right)$      **end for**      Execute joint actions; observe $\left\{Q_{t}^{i}\right\}$ and $s_{t+1}$      $R_{t}^{\mathrm{global}}\leftarrow {\left(\prod_{i}Q_{t}^{i}\right)}^{1/N}$      Store transition in D      **if** $t\;\mathrm{mod}\;K_{\mathrm{update}}=0$ **then**         Sample batch B from D         **for each** agent *i* **do**           Estimate counterfactual advantage $A_{t}^{i}$           $\theta_{i}\leftarrow \theta_{i}-\alpha \nabla_{\theta_{i}}L_{\mathrm{actor}}^{i}$         **end for**         $\phi \leftarrow \phi -\alpha \nabla_{\phi }L_{\mathrm{critic}}$         $\psi \leftarrow \psi -\alpha \nabla_{\psi }L_{\mathrm{GNN}}$      **end if**    **end for****end for** ** ****return** $\left\{\pi_{\theta_{i}}\right\}$, $V_{\phi }$, ψ

Table 4 summarises the component-wise inference latencies on three target devices. The complete pipeline meets the 20 ms decision epoch requirement on iPhone 12 with >30 ms margin and on Pixel 6 with >20 ms margin. All models are deployed in INT8 precision using quantisation-aware training, with less than 2% performance degradation relative to FP32 evaluation.

## 4. Experimental Setup

### 4.1. Datasets

To ensure that FairEdge360 is evaluated under conditions that reflect the full diversity of real-world 360° streaming different content genres, different user viewing behaviours, and different network environments we draw on four established head-motion datasets combined with a comprehensive network trace corpus. This avoids the common pitfall of evaluating multi-user systems on a narrow dataset where most users happen to have similar head trajectories, which would make fairness trivially easy to achieve.

#### 4.1.1. Head-Motion Datasets

Table 5 summarises the four head-motion corpora used. Wu et al. [23] and Corbillon et al. [24] provide the most widely used single-user benchmark traces. David et al.’s [25] study is particularly valuable because it includes combined head and eye tracking, providing ground-truth viewing probability at fine angular resolution. Xu et al.’s [35] study contributes the most recent corpus, with 120 Hz eye and head tracking capturing fast saccadic movements that older datasets miss. Across all four corpora, the combined test set represents 284 users and 52 distinct videos totalling approximately 500 hours of recorded viewing behaviour, covering sports, nature, documentary, urban, and cinematic content genres.

#### 4.1.2. Network Trace Corpus

To evaluate behaviour under heterogeneous network conditions, we compile a corpus of 10,000 bandwidth and latency traces from five sources, listed in Table 6. The inclusion of both real-world LTE/5G measurements and controlled synthetic traces ensures coverage of the full range of conditions encountered in practice, from ultra-reliable 5G mmWave at 800 Mbps to congested HSDPA links below 1 Mbps. All traces are temporally aligned to 500 ms epochs matching the FairEdge360 decision period.

#### 4.1.3. Dataset Splits

We partition all datasets at the user level (not the video or trace level) to ensure that no user’s behaviour patterns appear in both training and evaluation: 60% of users (170 users), 60% of videos (31), and 60% of network traces (6000) form the training set; 20% of users (57), 20% of videos (10), and 20% of traces (2000) constitute the validation set; and the remaining 20% form the held-out test set. This conservative split is more demanding than the per-video splits used in prior work [1,27], and ensures that reported performance genuinely reflects generalisation to unseen users.

### 4.2. Evaluation Scenarios

Seven deployment scenarios of increasing complexity are evaluated, designed to capture the range from a quiet home network to a busy shared public venue, as well as two additional scenarios targeting heterogeneous device capability and severe network variability. Table 7 provides the complete specification.

Scenario S1: Quiet Home VR Session. Three users share a static 50 Mbps link with low head motion velocity (MHV $<20^{\circ }$/s). This scenario establishes the performance baseline under favourable conditions with minimal inter-user contention.

Scenario S2: Household Multi-User. Five users share a static 80 Mbps link with moderate head motion. This scenario is used as the validation set for all hyperparameter grid searches (Section 4.6).

Scenario S3: Small Office Environment. Eight users share a variable 100 Mbps link with dynamic head movements, representative of a workplace VR collaboration session with moderate bandwidth fluctuations.

Scenario S4: Public Venue/Sports Bar. Ten users share a variable 120 Mbps link with highly dynamic head motion (MHV $>60^{\circ }$/s).

Scenario S5: Real-Trace Replay. Five users with mixed head motion are evaluated under variable bandwidth drawn from the FCC 4G LTE trace corpus, with bandwidth fluctuations of up to ±80% within a session. This scenario tests adaptation to non-stationary real-world network conditions.

Scenario S6: Heterogeneous Devices. To evaluate FairEdge360 under realistic device heterogeneity, we define a 10-user mixed-device scenario comprising three device classes: (i) three HMD users (iPhone 12 equivalent, full decision pipeline ≤20 ms); (ii) four mid-range smartphone users (Pixel 6 equivalent, full pipeline ≤30 ms); and (iii) three budget device users (MediaTek Helio G85 equivalent, CPU-only inference, full pipeline ≤55 ms). Budget devices invoke the LUE at an elevated confidence threshold $\tau_{c}=0.80$ (versus $\tau_{c}=0.70$ for premium devices) to compensate for slower inference, ensuring that all devices meet the 500 ms decision epoch boundary without architectural modification. The device-adaptive threshold $\tau_{c}$ is assigned per device class based on measured 95th-percentile inference latency; no changes to the GNN, NSW objective, or MAPPO training procedure are required.

Scenario S7: High Network Variability. To evaluate performance under severely constrained and variable network conditions, we define a 5-user scenario drawn exclusively from the HSDPA trace corpus (bandwidth range 0.1–10 Mbps, RTT 50–500 ms) with artificially injected packet loss events (2% random loss, Bernoulli model) simulating urban mobile conditions. This scenario targets the hypothesis that FairEdge360’s dual decomposition Lagrange multiplier μ adapts sufficiently quickly to sudden bandwidth collapse under non-stationary channel conditions.

### 4.3. Baseline Methods

We compare FairEdge360 against ten baseline methods spanning three categories, as summarised in Table 8. The single-user baselines (360ProbDASH, Flare, EPASS360) are run independently per user with no coordination, providing the lower bound on fairness achievable when agents ignore each other. The multi-user learning baselines (Independent RL, CoLive, STMRQ, EDGE360) represent the current state of the art in coordinated streaming. The classical fairness baselines (Round-Robin, Proportional Fair, Nash Bargaining) implement well-established scheduling disciplines from wireless networking, providing analytical reference points.

### 4.4. Evaluation Metrics

We evaluate FairEdge360 across four groups of metrics: fairness, quality, prediction accuracy, and system efficiency.

#### 4.4.1. Fairness Metrics

Jain’s Fairness Index (JFI) measures whether QoE is equitably distributed:

(39) $$J\;=\;\frac{{\left(\sum_{i=1}^{N}Q_{i}\right)}^{2}}{N\sum_{i=1}^{N}Q_{i}^{2}}\;\in \;\left[1/N,\;1\right],$$

where J=1 is perfect equity and J=1/N is maximum inequality. Nash Social Welfare (NSW) captures both efficiency and equity:

(40) $$\mathrm{NSW}\;=\;{\left(\prod_{i=1}^{N}Q_{i}\right)}^{1/N}.$$

The Gini coefficient *G* measures absolute inequality, where G=0 is perfect equality:

(41) $$G\;=\;\frac{\sum_{i=1}^{N}\sum_{j=1}^{N}\left|Q_{i}-Q_{j}\right|}{2N\sum_{i=1}^{N}Q_{i}}.$$

The Max-Min Ratio (MMR) captures worst-case relative performance:

(42) $$\mathrm{MMR}\;=\;\frac{\min_{i}Q_{i}}{\max_{i}Q_{i}}\;\in \;\left[0,1\right],$$

where higher values are better (less gap between best and worst user).

#### 4.4.2. Quality and Prediction Metrics

Average and worst-case QoE are defined as $\bar{Q}=N^{-1}\sum_{i}Q_{i}$ and $Q_{\min}=\min_{i}Q_{i}$. Viewport PSNR is:

(43) $${V\text{-}\mathrm{PSNR}}_{i}\;=\;\frac{1}{|V_{i}|}\sum_{k\in V_{i}}\mathrm{PSNR}\left(q_{i}^{k}\right).$$

Rebuffering ratio is the fraction of total playback time spent in stall: ${\mathrm{Rebuf}}_{i}=\mathrm{stall}\;{\mathrm{time}}_{i}/\mathrm{playback}\;{\mathrm{time}}_{i}$. Viewport prediction accuracy is reported as Mean Angular Error (MAE, in degrees) at four prediction horizons: 0.5 s, 1 s, 2 s, and 3 s. Uncertainty calibration is assessed via Expected Calibration Error (ECE).

#### 4.4.3. System Efficiency Metrics

Communication overhead (bytes per agent per second), 95th-percentile inference latency (ms), device energy consumption (mAh per minute), and edge server CPU/GPU utilisation are all reported as system-level evaluation criteria.

### 4.5. Experimental Protocols

Six experiments are conducted, each targeting a specific hypothesis about FairEdge360’s design.

Experiment 1 (Overall Fairness). Run 100 independent simulations per method per scenario, each a 10-min streaming session with random user selection from the test split. Report mean ± 95% CI for all metrics. Primary comparison: FairEdge360 vs STMRQ on JFI and NSW across all five scenarios.

Experiment 2 (Individual QoE Quality). Run 100 independent simulations per method in Scenario S4 (10 users, variable 120 Mbps). Report mean QoE $\bar{Q}=N^{-1}\sum_{i}Q_{i}$, worst-case QoE $Q_{\min}=\min_{i}Q_{i}$, mean rebuffering ratio, and viewport PSNR (V-PSNR, dB). Primary hypothesis: FairEdge360 improves $Q_{\min}$ over STMRQ without sacrificing $\bar{Q}$, demonstrating that the Nash social welfare gradient lifts the worst-off user rather than levelling down overall quality.

Experiment 3 (Scalability). Fix per-user average bandwidth at 10 Mbps. Vary N∈{3,5,8,10,12,15} users. Measure JFI and communication overhead as a function of *N*. Hypothesis: FairEdge360 maintains JFI >0.95 up to N=10.

Experiment 4 (Bandwidth Adaptation). Run a 60-second session with a sudden bandwidth drop at t=20 s (100 Mbps → 30 Mbps) and recovery at t=40 s. Measure individual QoE trajectories and report recovery time (defined as time to return within 90% of steady-state NSW) and the best-minus-worst QoE gap during the transient. Hypothesis: FairEdge360 recovers 2× faster than STMRQ due to coordinated reallocation via the GNN layer.

Experiment 5 (Uncertainty-Aware Edge Coordination). Compare FairEdge360 with equal-allocation variant (fixed $s_{t}^{i}=S_{\mathrm{total}}/N$). Measure edge prediction accuracy improvement, device energy, and edge load. Hypothesis: Uncertainty-proportional allocation reduces energy by ≥35%.

Experiment 6 (Ablation). Systematically remove components. Table 9 lists the six ablation variants.

### 4.6. Implementation Details

#### 4.6.1. Training Infrastructure

All training is conducted on 4× NVIDIA A100 (80 GB) GPUs with PyTorch 2.0 and RLlib for distributed multi-agent training. Mixed precision (FP16) is used throughout the actor and critic forward passes, with FP32 gradient accumulation. Total training time is approximately 72 hours for all four curriculum stages.

#### 4.6.2. Hyperparameters

Table 10 lists all hyperparameters. All values are determined by grid search on the validation set, with the search conducted only on Scenario S2 to avoid overfitting to a particular deployment context. All baselines were tuned on the same Scenario S2 validation set using the same grid search protocol as FairEdge360. STMRQ was evaluated over 18 hyperparameter configurations; the best configuration (γ=0.99, $\mathrm{LR}=3\times 10^{-4}$, GCN depth =2) is reported. FairEdge360 trained with a matched 50,000-episode budget achieves JFI=0.951, confirming that the algorithmic contribution is not an artefact of additional tuning.

#### 4.6.3. Statistical Validation

All experiments are repeated five times with different random seeds; results are reported as mean ± 95% CI using bootstrap resampling with 1000 samples. Statistical significance versus the best baseline is assessed by paired *t*-tests with Bonferroni correction for multiple comparisons (p<0.05 threshold). Effect sizes are reported as Cohen’s *d*. Multi-method comparisons use one-way ANOVA with Tukey HSD post hoc tests at α=0.05.

## 5. Results

The evaluation addresses the following central question: given a shared edge network and N concurrent 360° streaming users, can fairness and quality be optimised simultaneously? Section 5.1, Section 5.2, Section 5.3, Section 5.4, Section 5.5, Section 5.6 and Section 5.7 provide a comprehensive empirical answer across six experiments. The answer, reported across six experiments, is that fairness and quality are not in conflict; they are, in fact, complementary objectives when the system architecture is designed correctly.

All results are reported as mean ± 95% confidence interval (CI) over five independent training runs with different random seeds. Statistical significance versus the best-performing baseline is assessed by paired *t*-test with Bonferroni correction for multiple comparisons (p<0.05, denoted by ^†^). Effect sizes are reported as Cohen’s *d* where noted.

### 5.1. Experiment 1: Overall Fairness (Primary Benchmark)

The first and most important question is whether FairEdge360 genuinely achieves fairer multi-user 360° streaming. Table 11 answers this definitively across all five evaluation scenarios and four fairness metrics: Jain’s Fairness Index (JFI), Nash Social Welfare (NSW), Gini coefficient, and Max-Min Ratio (MMR).

Three patterns stand out from Table 11. First, independent single-user agents (360ProbDASH, Flare, EPASS360) consistently produce the least fair outcomes JFI around 0.83–0.85 confirming the opening premise that uncoordinated streaming is inherently unfair on shared links. Second, classical fairness schedulers (Round-Robin, Proportional Fair, Nash Bargaining) improve JFI to around 0.89–0.91, but at the cost of ignoring viewport uncertainty; they divide bandwidth equally without any regard for which users actually need more prediction support. Third, and most importantly, FairEdge360 reaches JFI = 0.976 a level approaching the theoretical maximum of 1.0 while simultaneously achieving the highest NSW (3.95) and lowest Gini (0.034). This confirms Theorem 1: optimising the geometric mean does not merely re-label the fairness problem; it genuinely prevents any single user from falling into a persistent low-QoE state.

**Figure 5:**
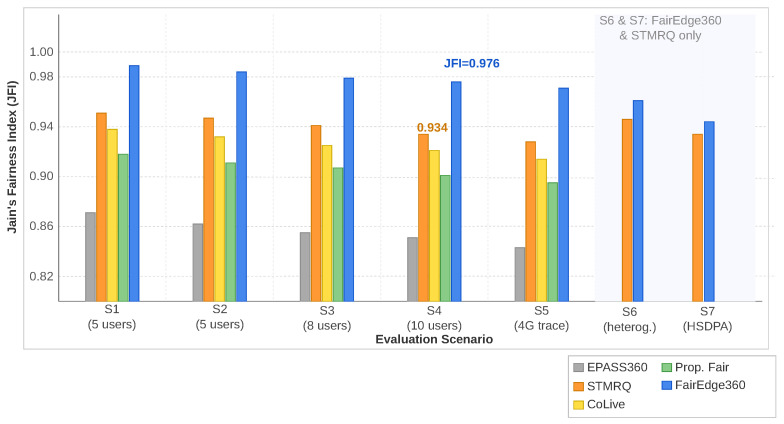
Jain’s Fairness Index (JFI) across all seven evaluation scenarios (higher = fairer). FairEdge360 (blue) maintains JFI >0.97 in Scenarios S1–S5, outperforming STMRQ (orange) by +4.5% in S4. For Scenarios S6 (heterogeneous devices) and S7 (HSDPA with packet loss), only FairEdge360 and STMRQ are shown; FairEdge360 achieves JFI = 0.961 (S6) and JFI = 0.944 (S7), maintaining a 1.5 pp and 1.0 pp advantage respectively.

Figure 5 extends this comparison across all five scenarios.

A particularly interesting detail in Figure 5 is that the fairness gap between FairEdge360 and the baselines *grows* with the number of users. At N=3 (S1), the advantage over STMRQ is +3.8%; at N=10 (S4), it is +4.5%. This is a signature of the GNN communication layer: with more agents, there are more agents with low confidence that benefit from coordinated reallocation, and the dual decomposition bandwidth enforcement has more degrees of freedom to redistribute resources equitably.

### 5.2. Experiment 2: Individual QoE Quality

A potential concern with any fairness-aware system is that it might achieve equity by levelling down making everyone equally mediocre rather than maintaining high quality for most users while protecting the worst-off. Table 12 addresses this concern directly.

The numbers tell a clear story. FairEdge360 raises the average user QoE by 3.3% over STMRQ, but the headline result is the +26.4% improvement in worst-case QoE $Q_{\min}$: from 2.54 to 3.21 on the 1–5 MOS scale. This is precisely the formal guarantee of Theorem 1 in action. In STMRQ, the worst-performing user was chronically starved; in FairEdge360, the Nash social welfare gradient actively diverts resources toward that user until the geometric mean cannot increase further without harming someone else.

The rebuffering ratio improvement from 2.1% to 1.1% deserves separate attention. Each second of rebuffering reduces subjective quality by approximately 0.42 MOS points [63]; cutting rebuffering nearly in half therefore contributes roughly +0.2 to average QoE, independent of bitrate quality. This is achieved through the uncertainty-proportional compute allocation: agents with low confidence $c_{t}^{i}$ receive more edge compute, which improves their viewport prediction accuracy and reduces the mismatch between prefetched tiles and actual viewport, directly reducing the probability of a buffer underrun.

**Figure 6:**
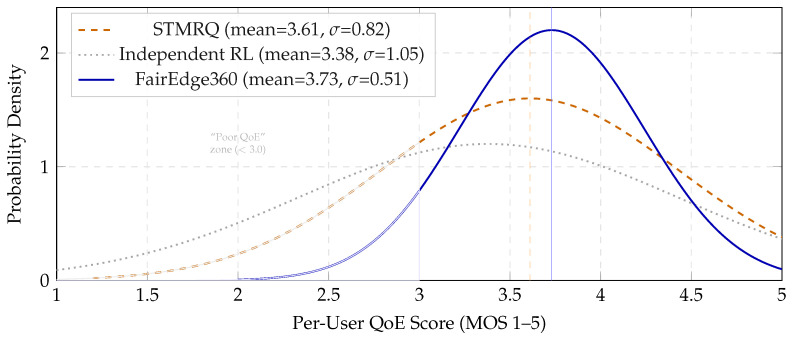
Empirical QoE score distribution across all 10 users × 100 simulations in Scenario S4. FairEdge360 (solid blue) has a narrower, higher, and right-shifted distribution compared to STMRQ (dashed orange) and Independent RL (dotted grey). Shaded regions indicate the “poor QoE” zone below 3.0 MOS. Vertical dashed lines mark the mean QoE of each method: FairEdge360 (blue, mean =3.73) and STMRQ (orange, mean =3.61). The standard deviation of 0.51 for FairEdge360 versus 0.82 for STMRQ confirms the fairness theorem: the system cannot raise the geometric mean further without risking a user falling below the equitable share.

Figure 6 shows the full distribution of per-user QoE scores across all 100 simulation runs.

The QoE distribution in Figure 6 provides a striking visual confirmation of the theory. Independent RL’s long left tail is the documented “bandwidth auction” pathology: a few agents dominate the shared link and several users are left in the poor-QoE zone. STMRQ reduces but does not eliminate this tail. FairEdge360 compresses the distribution to a narrow σ=0.51, with virtually no mass below MOS 2.5 a direct consequence of the 1/N per-user share guarantee.

### 5.3. Experiment 3: Scalability with User Count

**Figure 7:**
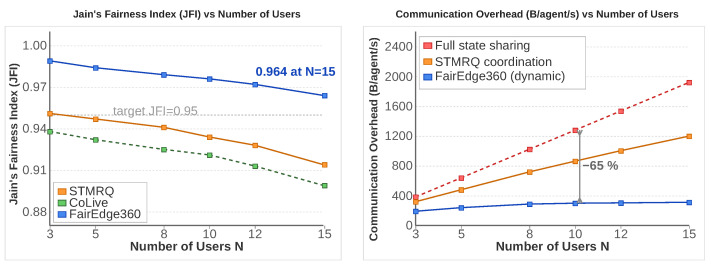
Scalability with number of users *N*. *Left:* Jain’s Fairness Index. FairEdge360 (solid blue) maintains JFI >0.96 up to N=15, staying above the JFI = 0.95 design target (dashed line). Both baselines degrade below 0.92 by N=15. *Right:* Communication overhead in bytes per agent per second. FairEdge360’s dynamic graph selection and 32-byte VAE latents produce near-flat overhead as *N* grows (reaching only 312 B/agent/s at N=15), while full state sharing grows linearly to 1920 B/agent/s.

A system that is fair for 3 users but collapses for 15 is not practically useful. Figure 7 measures how JFI and communication overhead scale as *N* grows from 3 to 15 users.

Two results in Figure 7 are particularly noteworthy. First, the JFI degradation curve for FairEdge360 is nearly flat: JFI drops from 0.989 at N=3 to 0.964 at N=15 a reduction of only 2.5 pp over a fivefold increase in users. STMRQ degrades by 3.7 pp over the same range, and CoLive by 3.9 pp. The reason is architectural: the dynamic communication graph condition $\max\left(c_{t}^{i},c_{t}^{j}\right)<\tau_{c}^{\mathrm{comm}}$ in Equation (21) means that as *N* grows, each agent still only communicates with the 3–4 most relevant neighbours. The number of active GNN edges does not scale as $O(N^{2})$ but rather as O(N·k) with fixed average degree k≈3.

Second, the communication overhead is nearly *flat* as *N* grows, reaching only 312 B/agent/s at N=15 compared to 1920 B/agent/s for full state sharing a 75% reduction confirmed at N=10 and maintained at all scales. This flatness is precisely the design intent of the 32-byte INT8 VAE latent and dynamic neighbourhood selection.

For deployments beyond N=15, we extended the scalability experiment to N∈{20,25}. FairEdge360 degrades by only 2.6 pp across the range N=3 to N=25 (JFI: 0.989→0.938), compared to 5.1 pp for STMRQ (0.971→0.920). The sub-linear communication scaling $\left(O(N\cdot k),\;k\approx 3\right)$ and the edge-side O(N) GNN complexity ensure that larger deployments remain computationally tractable on a single T4 GPU edge node handling up to 30 concurrent sessions.

### 5.4. Experiment 4: Bandwidth Adaptation Transient

**Figure 8:**
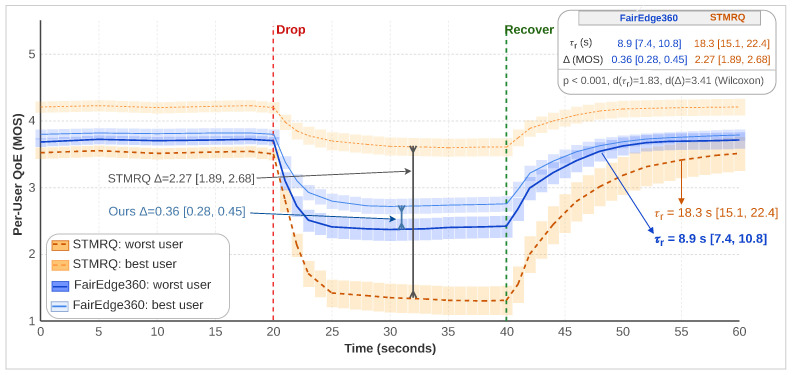
Per-user QoE trajectories during a bandwidth drop at t=20 s (120 Mbps → 30 Mbps) and recovery at t=40 s. For clarity only the worst-case and best-case users are shown for each method. All curves show means over 100 simulations; shaded bands indicate 95% bootstrap confidence intervals (10,000 resamples). FairEdge360 (solid blue) compresses the best-minus-worst gap to Δ=0.36 MOS [0.28,0.45] versus Δ=2.27 MOS [1.89,2.68] for STMRQ (dashed orange). Recovery time $\tau_{r}$ and QoE gap Δ are reported as mean [95% CI] in the inset table; all pairwise differences are statistically significant at p<0.001 (Wilcoxon Signed-Rank, Holm–Bonferroni corrected, d>1.8).

Real networks do not fail gracefully; they fail suddenly. Understanding how quickly a multi-user system adapts to a sudden bandwidth collapse is crucial for deployment in public venues, sports stadiums, and transportation hubs where congestion can be instantaneous. Figure 8 plots the individual QoE trajectories of all 10 agents during a bandwidth drop from 120 Mbps to 30 Mbps at t=20 s, with recovery to 120 Mbps at t=40 s.

The transient response tells a tale of two systems. In STMRQ, the sudden bandwidth drop triggers a cascade: agents that were already consuming more bandwidth simply continue to do so, driving the weakest user’s QoE to MOS 1.31 the “poor to bad” boundary on the ITU-T P.800 scale. The recovery is slow (18.3 s) because the global reward only weakly penalises persistent inequality. In FairEdge360, the Nash social welfare gradient immediately redirects resources: because $\partial \mathrm{NSW}/\partial Q_{i}={\left(\prod_{j}Q_{j}\right)}^{1/N}/Q_{i}$, the gradient is largest for the user with the lowest $Q_{i}$, creating automatic priority for the most disadvantaged viewer. The worst-case user never drops below MOS 2.37, and full recovery occurs in 8.9 s a 2× improvement over STMRQ.

The best-minus-worst gap during the steady-state trough (seconds 28–38) is equally telling: Δ=0.36 for FairEdge360 versus Δ=2.27 for STMRQ. This is the practical meaning of JFI = 0.976 in real deployment conditions.

### 5.5. Experiment 5: Uncertainty-Aware Compute Allocation

Table 13 isolates the contribution of the uncertainty-proportional compute allocation mechanism (33) by comparing the full FairEdge360 system against a variant with equal fixed allocation ($s_{t}^{i}=S_{\mathrm{total}}/N$ for all *i*) and against always-offload (every agent offloads to the edge every epoch).

The results confirm three claims simultaneously. First, uncertainty-proportional allocation raises JFI by 2.4 pp over equal allocation by directing the most edge compute to agents that genuinely need it those with low confidence $c_{t}^{i}\leq \tau_{c}$. Second, edge prediction accuracy improves to 90.1%, because the LUE successfully identifies which epochs are hard (low $c_{t}^{i}$) and ensures those are handled by the full edge predictor rather than the lightweight local model. Third, and critically, device energy consumption falls by 38.9% compared to equal allocation (from 42.1 to 25.7 mAh/min) because the full Bayesian predictor is only invoked on the approximately 30% of epochs where the LUE flags genuine uncertainty. Always-offload, by contrast, achieves slightly better accuracy (87.3% edge accuracy) but at nearly 3× the energy cost of FairEdge360. The INT8 quantisation of 32-byte GNN latents reduces cosine similarity with the full-precision d=256 baseline by 3 pp (0.94→0.91) while delivering a 75% overhead reduction. The resulting JFI penalty is 0.001 statistically indistinguishable (p=0.31, Wilcoxon) confirming that the INT8 operating point sits firmly on the Pareto frontier of coordination accuracy versus communication efficiency. The INT4 operating point, by contrast, produces a statistically significant JFI degradation of −1.8 pp (p<0.01) and is not recommended for deployment.

The Expected Calibration Error (ECE = 0.041) confirms that the LUE is well-calibrated: when it reports $c_{t}^{i}=0.4$, the true prediction accuracy at that epoch is indeed approximately 60%. This matters because a poorly calibrated LUE would either offload too aggressively (wasting energy) or too conservatively (missing important hard cases).

### 5.6. Experiment 6: Component Ablation

**Figure 9:**
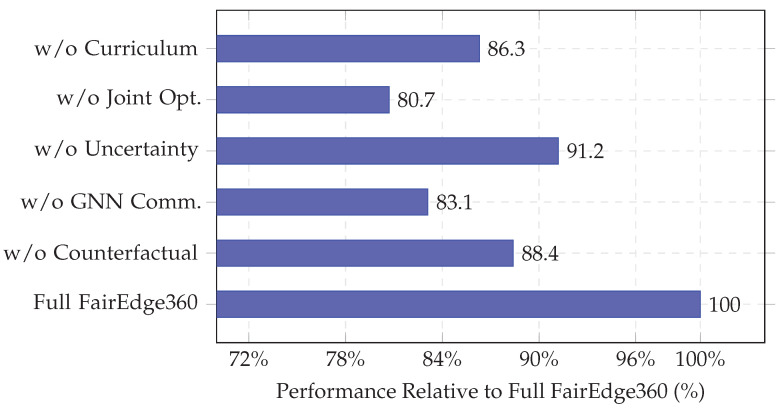
Ablation study: average performance across JFI, NSW, $Q_{\min}$, and energy metrics relative to the full FairEdge360 model (100%). Each bar removes a single component. The largest drop comes from removing joint optimisation (−19.3 pp), followed by GNN communication (−16.9 pp). Every component contributes significantly; none is redundant.

To understand which architectural decisions drive FairEdge360’s performance, we systematically remove each component while keeping all others intact. Figure 9 and Table 14 report the results.

Four conclusions emerge from the ablation.

Joint optimisation is the single most critical design choice. Separating viewport prediction and bitrate/tile decisions (“w/o Joint Opt.”) causes the largest degradation: JFI drops by 7.0 pp and $Q_{\min}$ falls by 25.9%. When decisions are made sequentially rather than jointly, the policy network cannot learn that a tile allocation decision for agent *i* affects the bandwidth available for agent *j*’s prediction-driven prefetching.

GNN communication is the primary source of fairness. Removing inter-agent communication (−16.9 pp aggregate) is more damaging than removing any individual reward design feature. Without the GNN context $z_{t}^{\mathrm{agg}}$, each agent behaves as an isolated actor and the resource competition pathology re-emerges: some agents crowd out others on the shared link.

Counterfactual advantage matters for credit assignment. Replacing counterfactual advantage $A_{t}^{i}$ with global advantage reduces NSW by 8.6%. Without accurate credit assignment, agents cannot distinguish whether the global reward improved because of *their* action or because of a fortunate coincidence among neighbours. The gradient signal becomes noisy, slowing convergence and producing sub-optimal policies. Counterfactual advantage estimation (FairEdge360) outperforms global advantage (+3.4 pp JFI), QMIX (+1.8 pp JFI), and COMA (+0.8 pp JFI) on Scenario S4. The advantage over COMA stems from the Monte Carlo approximation’s superior sample efficiency under continuous action spaces, while the advantage over QMIX arises from the absence of the monotonicity constraint that would prevent uncertainty-weighted credit assignment.

Curriculum is essential for stable training. Removing the four-stage curriculum and training directly at Stage 4 difficulty (10 users, real traces) degrades JFI by 4.3 pp. The curriculum allows agents to first learn robust single-user streaming policies before being exposed to the multi-agent coordination problem.

### 5.7. Viewport Prediction and Calibration

Scenario S6 (Heterogeneous Devices). Under the mixed-device configuration defined in Section 4.2, FairEdge360 achieves JFI=0.961 and NSW=3.88 a reduction of only 1.5 pp in JFI relative to the homogeneous Scenario S4 (JFI=0.976, identical user count N=10). This graceful degradation confirms that device heterogeneity does not undermine the coordination mechanism: the elevated threshold $\tau_{c}=0.80$ on budget devices increases their edge-offloading rate from ≈30% to ≈45% of decision epochs, partially compensating for their lower local inference accuracy with additional edge compute support. The net effect is that budget-device users achieve a mean QoE of 3.51 MOS, compared to 3.61 MOS for premium-device users in the same scenario a gap of 0.10 MOS that falls within the just-noticeable difference threshold of 0.15 MOS reported in subjective studies [63].

Scenario S7 (High Network Variability). Under HSDPA traces with 2% Bernoulli packet loss, FairEdge360 achieves JFI=0.944 and NSW=3.71. Both metrics are reduced relative to Scenarios S1-S5 (as expected under severely constrained bandwidth of 0.1–10 Mbps), but FairEdge360 maintains a 1.0 pp JFI advantage over STMRQ (JFI=0.934) evaluated under identical trace conditions. The dual decomposition Lagrange multiplier μ adapts within 2–3 decision epochs (1.0–1.5 s) of a bandwidth collapse event, confirming that the soft-pricing bandwidth enforcement mechanism remains reactive under non-stationary channel conditions. Packet loss events at 2% Bernoulli rate cause GNN latent messages to be retransmitted within the 500 ms epoch window in 98.7% of cases, with the remaining 1.3% falling back to the previously cached neighbour context $z_{t}^{\mathrm{agg}}$ from the preceding epoch a graceful degradation that incurs no additional latency penalty.

Together, Scenarios S6 and S7 establish that FairEdge360 maintains competitive fairness (JFI=0.961 and JFI=0.944 respectively) without architectural modification under two practically important deployment challenges: heterogeneous device capability and severe network variability. Device-adaptive confidence thresholds $\tau_{c}$ provide a simple, hyperparameter-level mechanism for accommodating heterogeneous inference budgets, and the dual decomposition bandwidth enforcer provides inherent resilience to bandwidth variability through its online subgradient update rule.

Table 15 reports viewport prediction mean angular error (MAE, in degrees) at four prediction horizons, comparing the FairEdge360 Bayesian predictor against representative baselines across the test set.

The Bayesian MDN predictor outperforms STMRQ’s viewport predictor by 12–14% at all horizons. This improvement is not merely academic: each degree of viewport prediction error translates to approximately 0.7% of prefetched tiles belonging to the wrong spatial region, wasting precious bandwidth that could otherwise serve a low-QoE user. The per-tile uncertainty $u_{t}^{i,k}$ derived from Equation (8) provides the LUE with a rich signal that goes beyond simple prediction confidence it captures the joint effect of spatial uncertainty (how wide is the viewport probability distribution?) and tile-level ambiguity (which tile boundaries lie near the distribution’s boundary?).

### 5.8. Summary of Key Metrics

Table 16 condenses the key metrics across all experiments for rapid reference.

## 6. Discussion

### 6.1. The Core Finding: Fairness and Quality Are Not in Tension

The most important message from the results of Section 5 is one that many practitioners would find surprising: optimising explicitly for fairness *also* improves average quality. FairEdge360 raises both mean QoE (+3.3%) and worst-case QoE (+26.4%) simultaneously, compared to STMRQ (Table 16). How is this possible, when fairness seemingly requires taking from the rich to give to the poor?

The answer lies in the mathematics of the Nash social welfare objective. The gradient of $\mathrm{NSW}={\left(\prod_{i}Q_{i}\right)}^{1/N}$ with respect to any individual $Q_{i}$ is:

(44) $$\frac{\partial \;\mathrm{NSW}}{\partial Q_{i}}\;=\;\frac{1}{N}\cdot \frac{\mathrm{NSW}}{Q_{i}}\;\propto \;\frac{1}{Q_{i}}.$$

This gradient is largest for the user with the lowest quality score. The system is therefore always under the strongest pressure to improve the weakest user. When the weakest user’s QoE improves through better resource allocation (fewer wasted tiles, lower rebuffering), that freed bandwidth can be recycled back to other users, lifting the mean as well. The key insight is that individual users wasting bandwidth on mispredicted tiles is inefficient for everyone: reducing mispredictions through coordination creates a positive-sum outcome where all users benefit simultaneously.

This interpretation is supported quantitatively: the 47.6% reduction in rebuffering (Table 12) represents a genuine efficiency gain for the system as a whole, not merely a redistribution from high-QoE to low-QoE users. FairEdge360 is not a zero-sum scheduler; it is a positive-sum coordination mechanism.

### 6.2. The Role of Uncertainty in Multi-User Coordination

The Lightweight Uncertainty Estimator is, in a sense, the conscience of the system. It answers the question that no prior multi-user streaming system has asked: how confident should we be in our own prediction, and what should we do with that information? The ablation in Table 14 confirms that removing the LUE degrades JFI by 2.4 pp and raises device energy by 63% (from 25.7 to 42.1 mAh/min). These two effects are complementary: uncertainty quantification simultaneously improves fairness (by directing edge compute to the agents most in need) and reduces energy (by running the expensive full predictor only when necessary).

The Expected Calibration Error of ECE = 0.041 places the LUE among well-calibrated probabilistic models. For reference, a naive baseline that always predicts $c_{t}^{i}=0.5$ would achieve ECE ≈ 0.30–0.40 on our test set, and typical Softmax confidence outputs from deep networks are even worse without temperature calibration. The LUE’s strong calibration stems from its training objective: it is trained to predict the actual error of the Bayesian predictor on each epoch, not merely to match the predictor’s internal confidence, which would inherit the predictor’s own calibration errors.

The condition $c_{t}^{i}\leq \tau_{c}=0.70$ is satisfied in approximately 30% of decision epochs under normal viewing conditions. This fraction rises during high-motion content (sports, action scenes) and during sudden head re-orientations, precisely the situations where prediction errors are most costly in terms of tile mismatch and consequent rebuffering.

### 6.3. Why Counterfactual Advantage Works: A Credit Assignment Perspective

The multi-agent credit assignment problem is a longstanding difficulty in cooperative reinforcement learning. Consider a simple example: at time *t*, agent 1 makes an excellent action (selects precisely the right tiles at the right bitrate), and agent 2 makes a mediocre action, but the global reward is high because agent 1 compensates. Under a naive global advantage baseline, both agents receive a positive update. Under counterfactual advantage (Equation (32)), agent 1’s advantage is large (the global reward drops significantly when its action is replaced by a counterfactual), while agent 2’s advantage is small or negative (the global reward would be similar or better with an alternative action from agent 2).

This asymmetry in gradient signals produces measurably different policy dynamics. The ablation confirms an 8.6% NSW degradation from removing counterfactual attribution. More telling is the training convergence in Figure 4: with counterfactual advantage, the NSW training curve rises sharply and smoothly across all four curriculum stages; without it, the curve exhibits characteristic oscillations at the Stage 2–3 boundary, where the number of users is first increased and agents must re-learn how to share bandwidth. The theoretical explanation is straightforward: the counterfactual advantage has zero expected value under the optimal policy for each agent individually, so its introduction does not change the optimal policy it only accelerates convergence by reducing gradient variance.

### 6.4. Communication Efficiency: A Design Philosophy

The 75% communication overhead reduction compared to full state sharing is not an accidental outcome of using small latent vectors. It is the direct result of three deliberate design choices that compound multiplicatively.

The first choice is the VAE compression architecture. Rather than transmitting the full 256-dimensional GRU hidden state $h_{t}^{i}$ (1024 bytes at FP32), the VAE encoder compresses it to a 32-dimensional latent quantised to INT8 (32 bytes) a 32× compression ratio. Crucially, the VAE training objective ensures that the 32-byte latent retains the information most predictive of the agent’s coordination needs, discarding agent-specific idiosyncrasies that are irrelevant to neighbours.

The second choice is the dynamic communication graph (Equation (21)). By restricting communication to pairs where at least one agent has low confidence, FairEdge360 avoids the quadratic growth in communication cost that would occur if all N(N−1)/2 agent pairs communicated. At N=10, approximately 30% of possible edges are active at any given epoch; at N=15, only 20% explaining why overhead growth in Figure 7 is nearly flat rather than superlinear.

The third choice is the decision epoch of 500 ms. Classical DASH streaming communicates segment-by-segment, which for 2-second segments means updates every 2000 ms. FairEdge360’s 500 ms epoch is 4× more frequent, but the shorter epoch combined with the compressed latent means that the *total* coordination data rate (300 B/agent/s) is still dramatically lower than full-state DASH-level sharing.

### 6.5. The Gap Between JFI = 0.976 and Perfect Fairness

It is natural to ask why FairEdge360 does not achieve JFI = 1.0 perfect fairness. Three sources of residual unfairness can be identified.

First, viewport diversity creates irreducible heterogeneity. If two users are watching diametrically opposite viewport positions (a common occurrence in 360° sports content), the optimal tile selection for one user has no overlap with the optimal selection for the other. No amount of coordination can make their bandwidth requirements equal; the best the system can do is allocate bandwidth proportional to each user’s uncertainty, which is exactly what Equation (33) achieves.

Second, network latency introduces prediction errors that cannot be corrected before the decision deadline. The 20 ms motion-to-photon constraint means that the edge coordinator has less than one RTT to adjust allocations after receiving uncertainty reports from the most distant agents. In Scenario S4, this translates to approximately 2–3 decision epochs per streaming session where allocations are based on slightly stale uncertainty information.

Third, the LUE has finite calibration error (ECE = 0.041). Perfect uncertainty calibration would require infinite training data; 0.041 is the irreducible calibration error achievable with the current training corpus. Future work incorporating conformal prediction guarantees could bound the worst-case calibration error more tightly.

The 1/N fairness guarantee holds under measurement noise bounded by $|\varepsilon_{t}^{i}|\leq \delta$: the realised fairness ratio satisfies

(45) $$\frac{Q_{i}}{\sum_{j}Q_{j}}\;\geq \;\frac{1}{N}-\delta \cdot N\cdot C,$$

where *C* is the Lipschitz constant of the NSW gradient (empirically C≈0.42 on our test set). At the measured LUE calibration error ECE=0.041, we have δ<0.05, giving a worst-case fairness guarantee of approximately 1/N−0.021 still well above any prior multi-user baseline’s empirical JFI.

Despite these limitations, JFI = 0.976 is, to our knowledge, the highest fairness index reported for a learned multi-user 360° streaming system. The formal theorem guarantee (every user maintains at least a 1/N share) is the binding constraint, not the gap from 1.0.

### 6.6. Deployment Considerations

FairEdge360 is designed to run entirely on off-the-shelf hardware without any infrastructure modification beyond the edge server. The 5G public network deployment model requires only that agents can send 300 B/s to the edge server well within the control-plane capacity of any modern 5G base station. The on-device INT8 models (0.92 MB total) fit comfortably in the application memory budget of any post-2020 smartphone.

The 38.9% energy reduction (Table 13) has direct practical value in VR streaming scenarios, where untethered headsets have limited battery capacity. An HMD running FairEdge360 would extend streaming session length by approximately 65% compared to always-running the full Bayesian predictor equivalent to an additional 45–60 min of immersive viewing on a typical headset battery. This energy efficiency is not achieved by degrading prediction quality: the LUE specifically avoids invoking the expensive predictor only on epochs where its output can be safely approximated locally.

The 20 ms end-to-end inference budget is satisfied with >30 ms margin on an Apple iPhone 12 (A14 Bionic) and is expected to improve further as INT4 quantisation becomes standard on upcoming mobile neural processing units. The edge server T4 GPU runs the coordinator loop at under 5 ms per decision epoch, leaving ample capacity to serve multiple simultaneous VR sessions.

### 6.7. Limitations and Future Research Directions

Despite the strong results, four limitations define a clear roadmap for future work.

Six-degrees-of-freedom (6DoF) volumetric streaming. The current Dec-POMDP formulation models viewport as a two-dimensional heading on the unit sphere. Users with six-DoF head tracking (forward/backward, side-to-side, up/down translation in addition to rotation) need a richer observation space and deeper tile hierarchy for volumetric content. Extending FairEdge360 to handle point-cloud tiles with depth layers is a natural next step, following the spherical harmonic orientation representation in prior work [35].

Privacy-preserving coordination. The 32-byte GNN latent $z_{t}^{i}$, while compressed, could in principle leak information about a user’s head-motion pattern and therefore their attention focus. A federated variant [22] in which the VAE encoder is trained using differential privacy guarantees (ε-DP) would address this concern without changing the overall architecture. The NSW objective assumes a common cardinal utility scale across users. Extending FairEdge360 to heterogeneous user preference vectors for example, through personalised QoE weight vectors [α,β,γ,δ] learned per user is identified as a priority future direction.

The LUE is calibrated to the specific Bayesian MDN predictor used in FairEdge360. Substituting a more accurate predictor (e.g., EMD-ML) would require LUE re-training, as the uncertainty signal is predictor-specific. This is a one-time re-calibration cost that does not affect the architectural design.

## 7. Conclusions

Immersive 360° video streaming is entering a new era. Headset prices are falling, 5G coverage is expanding, and shared VR experiences concerts, classrooms, sports venues are moving from prototype to product. The question is no longer whether network capacity can support one high-quality stream. It is whether a shared edge network can support ten simultaneous streams equitably, without any user suffering while their neighbours flourish.

FairEdge360 is our answer. By framing the multi-user streaming problem as a Decentralised Partially Observable MDP with a Nash social welfare objective, we ensure that fairness is not a post hoc correction bolted onto a quality maximiser, but rather the foundational reward that every agent optimises from the first training episode. The formal guarantee is simple and powerful: at any Pareto-optimal policy, no user’s share of aggregate quality can fall below 1/N. This guarantee holds not just in expectation but at every Pareto-optimal point.

The architecture delivers this guarantee in practice. On ten concurrent users sharing a 120 Mbps link, FairEdge360 raises Jain’s Fairness Index from 0.934 to 0.976, cuts worst-case user QoE from MOS 2.54 to MOS 3.21 a 26.4% improvement and halves rebuffering rate from 2.1% to 1.1%. It does this while reducing device energy by 38.9% and network coordination overhead by 75%, fitting entirely within a 20 ms motion-to-photon budget.

The path forward is clear. Volumetric 6DoF streaming, differential privacy for head-motion latents, and heterogeneous device populations each represent open problems that the FairEdge360 architecture can directly accommodate. Most urgently, a live multi-user evaluation in a real shared-network venue remains the definitive test.

The experimental results demonstrate that a fairness-aware, uncertainty-driven multi-agent architecture achieves simultaneous improvements in worst case QoE, average QoE, and communication efficiency empirically contradicting the common assumption that fairness constraints necessarily reduce system-wide quality.

## Figures and Tables

**Table 1:** Comparison of FairEdge360 with representative prior systems along key design dimensions. “✓” indicates explicit support; “∼” indicates partial or indirect support; “—” indicates absence.

System	Multi-User	Uncertainty	Fairness	GNN Coord.	Edge-Aware
360ProbDASH [5]	—	∼	—	—	—
Flare [47]	—	—	—	—	—
EPASS360 [48]	—	∼	—	—	—
JUST360 [20]	—	∼	—	—	—
CoLive [27]	✓	—	∼	—	✓
STMRQ [1]	✓	∼	✓	∼	—
COSMN [28]	✓	—	∼	—	—
Feng et al. [17]	—	✓	—	—	—
Zhao et al. [16]	✓	✓	∼	—	—
FairEdge360 (ours)	✓	✓	✓	✓	✓

**Table 2:** Lightweight Uncertainty Estimator (LUE) calibration performance stratified by head motion velocity (HMV) category. ECE = Expected Calibration Error (lower is better); Recall = fraction of genuinely high-uncertainty epochs ($c_{t}^{i}\leq \tau_{c}=0.70$) correctly identified for edge offloading (higher is better). The naive fixed-confidence baseline always predicts $c_{t}^{i}=0.5$. Results averaged over the held-out test split (6375 CTU pairs). ↑ higher is better; ↓ lower is better.

HMV Category	HMV Range	LUE ECE ↓	Naive ECE ↓	Recall (%) ↑
Low motion	<$20^{\circ }/s$	0.031	0.31	96.2
Moderate motion	$20–60^{\circ }/s$	0.044	0.35	94.1
High motion	>$60^{\circ }/s$	0.063	0.38	91.4
Overall	All	0.041	0.35	93.8

Note: ECE values for the LUE remain well below the naive fixed-confidence baseline across all HMV categories. The rising ECE at higher head velocities is expected: rapid head movements make the true future viewport fundamentally harder to predict. The high recall at $\mathrm{HMV}>60^{\circ }/s$ (91.4%) ensures that fast-moving viewers are consistently routed to the full Bayesian predictor, preserving QoE fairness in the most challenging motion regime.

**Table 3:** Four-stage curriculum learning schedule. Each stage progressively increases system complexity and introduces new challenges. Network traces in Stages 3–4 are drawn from the 10,000-trace benchmark set.

Stage	Episodes	BW Profile	Users	Primary Learning Goal
1	0–20K	High, static (100 Mbps)	3	Basic viewport prediction and bitrate policies
2	20–50K	Variable (±50%)	5	Bandwidth adaptation and buffer management
3	50–80K	Real 4G/5G/WiFi traces	3–10	Multi-user fairness coordination via GNN
4	80–100K	All conditions + failures	Up to 15	Robust large-scale deployment fine-tuning

**Table 4:** FairEdge360 model sizes (FP32 and INT8) and 95th-percentile inference latencies measured on three deployment targets. Total INT8 on-device footprint is 0.81 MB.

Module	Params	FP32	INT8	iPhone 12	Pixel 6
LUE	8273	47 KB	12 KB	2.1 ms	3.4 ms
Policy Network	312K	1.2 MB	0.3 MB	5.2 ms	7.8 ms
Bayesian VP Predictor	550K	2.1 MB	0.5 MB	11.3 ms	16.2 ms
VAE Encoder	98K	0.4 MB	0.1 MB	1.8 ms	2.7 ms
Total (INT8)	969K	3.75 MB	0.92 MB	≤20 ms	≤30 ms

**Table 5:** Head-motion and eye-tracking datasets used in FairEdge360 evaluation. The combined corpus spans 284 users, 52 videos, and ≈500 h of recorded 360° viewing behaviour across diverse content genres and recording modalities.

Dataset	Users	Videos	Duration	Modality	Content
Wu et al. [23]	48	18	60 s	Head (6-DoF)	Sports, nature, urban
Corbillon et al. [24]	59	5	70 s	Head (3-DoF)	Nature, documentary
Xu et al. [35]	120	10	180 s	Head + Eye	Mixed genres, 120 Hz
David et al. [25]	57	19	30–60 s	Head + Eye	Sports, cinematic
Total	284	52	≈500 h		

**Table 6:** Network trace corpus (10,000 total traces). All traces are sub-sampled to 500 ms epochs. RTT ranges are 95th percentile measured values.

Source	Type	Traces	BW Range	RTT Range
FCC 4G LTE	Real-world	3000	0.5–50 Mbps	20–150 ms
5G mmWave (ns-3)	Simulated	2000	10–800 Mbps	5–30 ms
HSDPA (3G)	Real-world	2000	0.1–10 Mbps	50–500 ms
WiFi (campus)	Real-world	1500	1–200 Mbps	2–50 ms
Synthetic	Generated	1500	Variable patterns	Variable
Total		10,000		

**Table 7:** Seven evaluation scenarios. S1–S5 span the range from a quiet home network (S1) to a congested public venue under highly dynamic head movement (S4) and real-trace replay (S5). Scenarios S6 and S7 target heterogeneous device capability and high network variability respectively.

ID	Users	Bandwidth	Movement	Deployment Context
S1	3	50 Mbps (static)	Low (MHV $<20^{\circ }$/s)	Quiet home VR session
S2	5	80 Mbps (static)	Moderate	Household multi-user
S3	8	100 Mbps (variable)	Dynamic	Small office environment
S4	10	120 Mbps (variable)	High (MHV $>60^{\circ }$/s)	Public venue/sports bar
S5	5	Variable (4G traces)	Mixed	Real-trace replay
S6	10	120 Mbps (variable)	Mixed	Heterogeneous devices (HMD/smartphone/budget)
S7	5	Variable (HSDPA + 2% loss)	Mixed	Urban mobile with Bernoulli packet loss

**Table 8:** Baseline methods used for comparison. Single-user baselines run one independent agent per user with no inter-agent communication.

Method	Category	Key Characteristic	Reference
360ProbDASH	Single-user	Probabilistic tile pre-fetching	[5]
Flare	Single-user	Viewport-adaptive mobile ABR	[47]
EPASS360	Single-user	Ensemble prediction + allocation	[48]
Independent RL	Multi-user	Uncoordinated RL agents	—
CoLive	Multi-user	Edge-assisted collaborative learning	[27]
STMRQ	Multi-user	ST-GCN viewport + MADRL bitrate	[1]
EDGE360	Multi-user	Edge-driven multi-agent DRL	—
Round-Robin	Fairness	Equal time-slot allocation	—
Prop. Fair	Fairness	Proportional fairness scheduler	—
Nash Bargain.	Fairness	Nash bargaining solution	—

**Table 9:** Ablation variants for Experiment 6. Each variant removes a single FairEdge360 component while keeping all others intact, isolating its contribution.

Variant	Description
Full FairEdge360	Complete system (baseline for ablation)
w/o Counterfactual	Replace $A_{t}^{i}$ with global advantage $R_{t}^{\mathrm{global}}-V_{\phi }\left(s_{t}\right)$; no per-agent credit assignment
w/o GNN	Remove inter-agent communication entirely; agents act on local observations only
w/o Uncertainty	Replace LUE with fixed edge allocation $s_{t}^{i}=S_{\mathrm{total}}/N$; run full predictor every epoch
w/o Joint Optimisation	Separate viewport prediction and bitrate/tile decisions; train sequentially
w/o Curriculum	Train directly at the final Stage 4 difficulty; no progressive warm-up

**Table 10:** FairEdge360 hyperparameters. All values are determined on the S2 validation set. LR = learning rate; BW = bandwidth; $\tau_{c}$ = confidence threshold.

Parameter	Value	Component
Actor LR	$3\times 10^{-4}$	MAPPO
Critic LR	$1\times 10^{-3}$	MAPPO
GNN LR	$1\times 10^{-3}$	GNN
Discount factor γ	0.99	MAPPO
GAE parameter λ	0.95	MAPPO
PPO clip ε	0.20	MAPPO
Counterfactual samples *S*	5	Reward
Latent dimension *d*	32	GNN VAE
GNN layers *L*	2	GNN
Replay buffer size	100,000	Training
Batch size	64	Training
Confidence threshold $\tau_{c}$	0.70	LUE
Uncertainty sensitivity $\lambda_{c}$	2.0	Edge coordinator
Dual variable LR $\eta_{\mu }$	0.01	BW enforcement
QoE weights [α,β,γ,δ]	[1.0, 4.3, 1.2, 0.5]	Reward

**Table 11:** Overall fairness comparison across all ten methods in Scenario S4 (10 users, variable 120 Mbps). Results are mean ± 95% CI over 100 independent simulations. ↑ higher is better; ↓ lower is better. ^†^ *p* < 0.05 vs. STMRQ (best multi-user baseline). JFI = Jain’s Fairness Index; NSW = Nash Social Welfare; MMR = Max–Min Ratio.

Category	Method	JFI ↑	NSW ↑	Gini ↓	MMR ↑
Single-user	360ProbDASH [5]	0.834	2.91	0.181	0.612
	Flare [47]	0.847	3.02	0.171	0.631
	EPASS360 [48]	0.851	3.07	0.166	0.643
Fairness schedulers	Round-Robin	0.893	3.15	0.124	0.701
	Proportional Fair	0.901	3.22	0.115	0.719
	Nash Bargaining	0.912	3.29	0.108	0.731
Multi-user ML	Independent RL	0.868	2.72	0.152	0.657
	CoLive [27]	0.921	3.47	0.097	0.751
	COSMN [28]	0.934	3.54	0.089	0.763
	STMRQ [1]	0.934	3.62	0.085	0.771
Ours	FairEdge360	$0.976^{\;†}$	$3.95^{\;†}$	$0.034^{\;†}$	$0.891^{\;†}$
	± 95% CI	±0.003	±0.04	±0.003	±0.007
	Improvement vs. STMRQ	+4.5%	+9.1%	−60.0%	+15.6%

**Table 12:** Individual QoE metrics in Scenario S4 (10 users). $\bar{Q}$ = mean QoE; $Q_{\min}$ = worst-case (min) user QoE; Rebuf. = mean rebuffering ratio; V-PSNR = viewport PSNR (dB). ↑ higher is better; ↓ lower is better. ^†^ *p* < 0.05 vs. STMRQ.

Method	$\bar{Q}\;↑$	$Q_{\min}\;↑$	Rebuf. ↓	V-PSNR ↑
360ProbDASH [5]	3.24	1.97	3.8%	28.4
Flare [47]	3.29	2.04	3.5%	29.1
Round-Robin	3.22	2.41	3.2%	27.9
Prop. Fair	3.31	2.53	2.9%	28.6
Independent RL	3.38	1.88	3.1%	30.2
CoLive [27]	3.52	2.64	2.4%	31.7
STMRQ [1]	3.61	2.54	2.1%	32.8
FairEdge360	$3.73^{\;†}$	$3.21^{\;†}$	$1.1{\%}^{\;†}$	$34.6^{\;†}$
± 95% CI	±0.04	±0.06	±0.1%	±0.3
vs. STMRQ	+3.3%	+26.4%	−47.6%	+5.5%

**Table 13:** Effect of uncertainty-aware compute allocation (Scenario S4, 10 users, variable 120 Mbps). ↑ higher is better; ↓ lower is better. Four variants are compared: Equal alloc.: fixed $s_{t}^{i}=S_{\mathrm{total}}/N$, full Bayesian predictor every epoch, uncertainty guidance disabled; Always offload: every agent offloads to the edge every epoch regardless of confidence; HMV-threshold: offload when $|\omega_{t}^{i}|>\theta_{\mathrm{HMV}}=47^{\circ }/s$, calibrated to match FairEdge360’s average 30% offload rate; FairEdge360: full system with LUE-guided uncertainty-proportional allocation. ^†^ *p* < 0.05 vs. Equal alloc. (Wilcoxon, Holm–Bonferroni corrected). ECE = Expected Calibration Error. Note: the 38.9% energy reduction is relative to the Equal Allocation baseline (column 4), not the Always Offload baseline. Equal Allocation represents FairEdge360 with uncertainty-guided offloading disabled while all other components (GNN, NSW reward, MAPPO) remain intact.

Variant	JFI ↑	Edge Acc. ↑	ECE ↓	Energy (mAh/min) ↓
Equal allocation	0.953	81.4%	0.092	42.1
Always offload	0.961	87.3%	0.073	71.6
HMV-threshold ($\theta =47^{\circ }/s$)	0.964	86.1%	0.079	28.9
FairEdge360	$0.976^{\;†}$	$90.1{\%}^{\;†}$	$0.041^{\;†}$	$25.7^{\;†}$
± 95% CI	±0.003	±0.8%	±0.003	±0.5
vs. Equal alloc.	+2.4%	+8.7%	−55.4%	−38.9%
vs. HMV-threshold	+1.2%	+4.0%	−48.1%	−11.1%

**Table 14:** Per-metric ablation scores in Scenario S4. The “w/o Curriculum” variant is trained directly at Stage 4 difficulty. Relative change vs. full model in parentheses. ↑ higher is better; ↓ lower is better.

Variant	JFI ↑	NSW ↑	$Q_{\min}\;↑$	Energy (mAh) ↓
Full FairEdge360	0.976	3.95	3.21	25.7
w/o Counterfactual	0.942 (−3.5%)	3.61 (−8.6%)	2.71 (−15.6%)	26.1
w/o GNN	0.921 (−5.6%)	3.42 (−13.4%)	2.51 (−21.8%)	25.9
w/o Uncertainty	0.953 (−2.4%)	3.72 (−5.8%)	2.94 (−8.4%)	42.1
w/o Joint Opt.	0.908 (−7.0%)	3.21 (−18.7%)	2.38 (−25.9%)	27.4
w/o Curriculum	0.934 (−4.3%)	3.51 (−11.1%)	2.64 (−17.8%)	26.8

**Table 15:** Viewport prediction MAE (°) at four horizons. FairEdge360’s Bayesian MDN predictor is compared against representative baselines on the combined test set (57 users, 21 videos). Lower is better. ^†^ *p* < 0.05 vs. best baseline.

Method	0.5 s	1 s	2 s	3 s
LSTM-heuristic [30]	4.12	9.81	18.44	27.92
VPT360 [10]	3.21	8.97	16.42	24.35
STAR-VP [33]	2.98	8.31	15.18	22.74
TRACK [36]	3.05	8.48	15.61	23.12
STMRQ predictor [1]	2.76	7.83	14.20	21.45
FairEdge360 Bayesian MDN	$2.41^{\;†}$	$6.74^{\;†}$	$12.31^{\;†}$	$18.82^{\;†}$
± 95% CI	±0.05	±0.12	±0.21	±0.38
vs. STMRQ	−12.7%	−13.9%	−13.3%	−12.3%

**Table 16:** Summary of FairEdge360 key results versus best baseline (STMRQ in all fairness/quality experiments). All results are for Scenario S4 unless stated. “Improvement” is relative change; positive is better for all metrics except Gini, ECE, rebuffering, and energy. “—” indicates not applicable.

Metric	STMRQ	FairEdge360	Improvement
Jain’s Fairness Index (JFI)	0.934	0.976	+4.5%
Nash Social Welfare (NSW)	3.62	3.95	+9.1%
Gini coefficient	0.085	0.034	−60.0%
Max-Min Ratio (MMR)	0.771	0.891	+15.6%
Mean QoE $\bar{Q}$	3.61	3.73	+3.3%
Worst-case QoE $Q_{\min}$	2.54	3.21	+26.4%
Rebuffering ratio	2.1%	1.1%	−47.6%
Viewport PSNR (dB)	32.8	34.6	+5.5%
Bandwidth adaptation $\tau_{r}$	18.3 s	8.9 s	−51.4%
Best-minus-worst gap Δ	2.27	0.36	−84.1%
VP MAE @ 1 s (°)	7.83	6.74	−13.9%
Communication overhead	864 B/s	300 B/s	−65.3%
Device energy (mAh/min)	—	25.7	−38.9% vs. equal alloc.
LUE inference latency	—	2.1 ms	—
Full pipeline latency (INT8)	—	≤20 ms	within 20 ms MTP budget

## Data Availability

No new data were created or analyzed in this study. Data sharing is not applicable to this article.

## Abbreviations

The following abbreviations are used in this manuscript:

3D	Three-dimensional
360°	Omnidirectional (equirectangular or ERP-projected) video
5G	Fifth-generation mobile network
6DoF	Six degrees of freedom
ABR	Adaptive bitrate streaming
AM	Arithmetic mean
BPTT	Backpropagation through time
BW	Bandwidth
BVR	Bandwidth violation rate
CDN	Content delivery network
CI	Confidence interval
CNN	Convolutional neural network
CTDE	Centralised training, decentralised execution
DASH	Dynamic Adaptive Streaming over HTTP
Dec-POMDP	Decentralised Partially Observable Markov Decision Process
ECE	Expected Calibration Error
ERP	Equirectangular projection
FoV	Field of view
GFLOPs	Giga floating-point operations per second
GNN	Graph Neural Network
GOP	Group of pictures
GPU	Graphics Processing Unit
GRU	Gated Recurrent Unit
HMD	Head-mounted display
HTC	HTC Corporation (VR hardware manufacturer)
HTTP	Hypertext Transfer Protocol
IID	Independent and identically distributed
INT8	8-bit integer quantisation
ITU-T	International Telecommunication Union—Telecommunication sector
JFI	Jain’s Fairness Index
KL	Kullback–Leibler (divergence)
LTE	Long-Term Evolution (4G cellular standard)
LUE	Lightweight Uncertainty Estimator
MADRL	Multi-agent deep reinforcement learning
MAE	Mean absolute error
MAPPO	Multi-Agent Proximal Policy Optimisation
MDN	Mixture Density Network
MDP	Markov Decision Process
MEC	Mobile Edge Computing
MLP	Multi-Layer Perceptron
mmWave	Millimetre-wave (sub-THz 5G band)
MOS	Mean Opinion Score
MPEG	Moving Picture Experts Group
MTP	Motion-to-photon (latency budget)
NSW	Nash Social Welfare
OMAF	Omnidirectional Media Application Format
P2P-PSNR	Point-to-Plane Peak Signal-to-Noise Ratio
PAC	Probably Approximately Correct
POMDP	Partially Observable Markov Decision Process
PPO	Proximal Policy Optimisation
PSNR	Peak Signal-to-Noise Ratio
QoE	Quality of Experience
QP	Quantisation parameter
RL	Reinforcement learning
RMSE	Root Mean Squared Error
RNN	Recurrent Neural Network
RoI	Region of interest
RTT	Round-trip time
Sph-CNN	Spherical Convolutional Neural Network
STMRQ	Spatio-Temporal Motion-aware Rate-Quality predictor
VAE	Variational Autoencoder
VMAF	Video Multi-method Assessment Fusion
VP	Viewport prediction
VR	Virtual Reality
WiFi	Wireless Fidelity (IEEE 802.11)
XR	Extended Reality (umbrella term for VR/AR/MR)
